# The p53 Super-Hub Architecture: From Planar to Volumetric Network Pharmacology

**DOI:** 10.3390/ijms27219752

**Published:** 2026-10-30

**Authors:** Daniil R. Bazanov

**Affiliations:** 1 Department of Chemistry, Shenzhen MSU-BIT University, Shenzhen 518172, China; daniil_bazanov@mail.ru; 2 Department of Chemistry, M. V. Lomonosov Moscow State University, 119991 Moscow, Russia

**Keywords:** p53 network, systems pharmacology, synthetic lethality, translational oncology, super-hub protein, allosteric regulation, evolutionary adaptations

## Abstract

The p53 protein is a central tumor suppressor; however, its status as an intracellular super-hub renders direct pharmacological reactivation via the traditional “lock-and-key” paradigm highly challenging due to systemic toxicity and the evolutionary plasticity of tumor networks. To address this translational barrier, we apply a systems engineering approach, deconstructing the p53 signaling architecture into seven hierarchical levels. By mapping current therapeutic strategies onto this framework, we illustrate that no ideal single-target strategy currently exists. While existing alternative approaches—such as activating redundant homologs, allosteric regulation, or exploiting synthetic lethality (e.g., WEE1 inhibition)—provide valuable bypass tactics, they frequently remain insufficient in isolation. Instead, we propose that a genuine therapeutic solution requires a comprehensive, multi-level intervention strategy that simultaneously modulates several layers of the network. We hypothesize that this concept of distributed signal load is biologically supported by natural evolutionary models: increased TP53 copy numbers in elephants and multi-tiered contact inhibition in naked mole-rats suggest the efficacy of peripheral network offloading. Ultimately, we advocate a conceptual shift from planar mono-targeting toward adaptive network management, aiming to identify tissue-specific local optima for coordinated multi-node interventions.

## 1. Introduction

The p53 protein serves as a central tumor suppressor, with mutations identified in over half of all human cancers. However, despite decades of research, the clinical translation of p53-targeted therapies remains a formidable challenge. For instance, while highly potent small-molecule inhibitors designed to block the MDM2-p53 protein–protein interaction have advanced to clinical trials, their efficacy is frequently limited by acquired resistance and dose-limiting toxicity in normal tissues [1]. To overcome these pharmacological limitations, recent advances in structural biology suggest moving beyond classical structural hierarchies to analyze proteins as interconnected networks of coevolving, quasi-independent amino acid “sectors” [2,3]. To understand the fundamental complexity of this challenge, this review draws direct parallels between molecular biology, computer science, and complex network theory.

The primary objective of our work is to deconstruct the p53 architecture into hierarchical levels—ranging from local post-translational modifications to global tissue homeostasis—and to evaluate existing therapeutic approaches against this framework. By projecting pharmacological strategies onto this multilevel structure, we demonstrate why network bypass routes, such as allosteric regulation, the exploitation of synthetic lethality, and the utilization of evolutionary reserves possess significantly greater therapeutic potential than direct reactivation attempts. Furthermore, we discuss how the viability of this multilevel distribution concept is convincingly validated by natural evolutionary models. Building on these insights, this review emphasizes the critical need to transition toward adaptive network management for effective cancer therapy.

## 2. Evolution of p53 and Its Regulation

To fully comprehend the p53 network, it is essential to examine its evolutionary history. Several comprehensive studies have addressed the emergence and evolution of the p53 protein. Notable among these are the works by Belyi et al. [4], Aberg [5], Nidelcu [6], and Joerger [7]. Arnold Levine, the discoverer of p53, provides an essential synthesis of the research progress in this area [8]. The p53 regulators MDM2 and MDM4 (MDMX) have received comparatively less attention from an evolutionary standpoint; nevertheless, the studies by Momand [9] and the aforementioned work by Aberg [5] are particularly noteworthy. This section aims to briefly demonstrate the significance of this genome integrity regulator from an evolutionary perspective. Why and when did it become critical to control genome integrity? When did the specific regulation of p53 emerge? And what drove the evolutionary divergence of p53 and MDM2 homologs?

**Figure 1:**
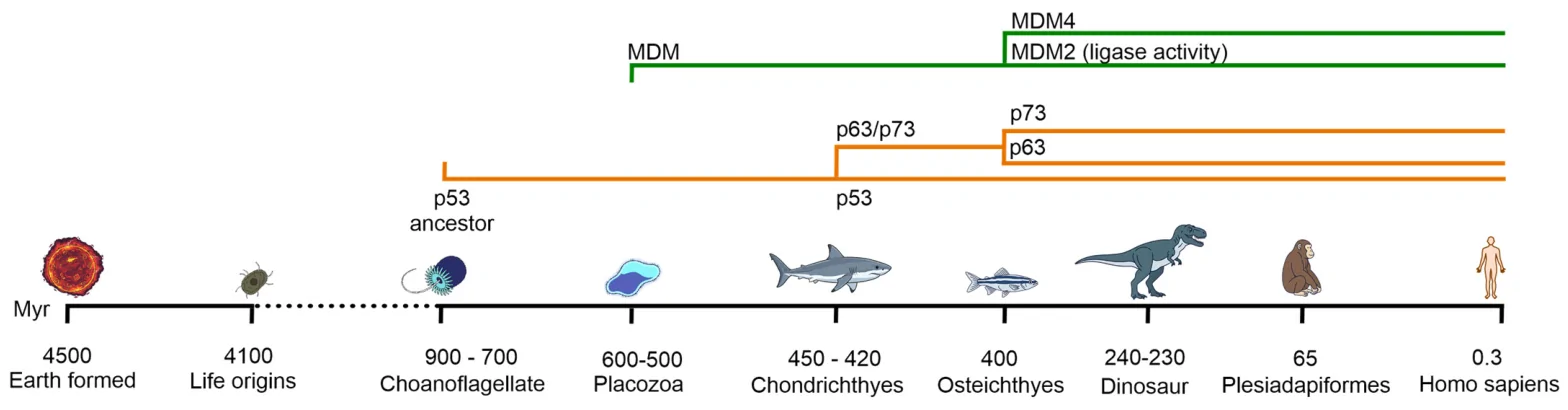
Evolutionary timeline of the p53 protein family and its MDM regulators. The phylogenetic tree illustrates the emergence of the p53 ancestor in early organisms (e.g., choanoflagellates, 900–700 Myr ago) and its subsequent genetic duplications. The initial divergence into independent p53 and p63/p73 lineages occurred in cartilaginous fishes (*Chondrichthyes*, 450–420 Myr ago) alongside the development of complex multicellularity. A second duplication in bony fishes (*Osteichthyes*, ~400 Myr ago) established the distinct p63 and p73 (denoted in some models as an ancestral p83 variant) genes. The parallel evolution of the MDM ancestor and its divergence into MDM2 (featuring ligase activity) and MDM4 reflects the increasing complexity and necessary fine-tuning of the p53 regulatory network.

From an evolutionary perspective, the p53 ancestor—which more closely resembles p63 and p73—first emerged in choanoflagellates (unicellular organisms) and sea anemones (multicellular organisms) [4,6,9] (Figure 1). In the latter, this ancestral protein functions to monitor DNA duplication in germ cells [4]. As cellular organization increased in complexity and cell differentiation evolved, it became necessary to distribute these regulatory functions across distinct proteins. Consequently, in cartilaginous fishes, the p53 ancestor diverged into two separate genes: p53 and p63/p73. This divergence was driven by a fundamentally new paradigm of multicellular existence [4]. From this evolutionary point forward, multicellular organisms developed distinct organs and tissues that required lifelong continuous renewal via somatic stem cells. Therefore, the newly independent p53 gene acquired the role of “guardian of the genome” to strictly regulate somatic stem cell division [4]. With further organismal complexity in bony fishes, the p63/p73 gene underwent another duplication, separating into independent p63 and p73 genes to enable finer control over tissue differentiation. Specifically, p63 became responsible for epithelial tissues, while p73 assumed the regulation of immunological and neuronal development [4].

The necessity for specific regulation of cell death arises during the evolutionary transition from unicellular to multicellular organisms; in primitive unicellular life forms, the p53 ancestor is likely regulated by non-specific ubiquitin ligases [10,11]. The MDM ancestor is first observed in *Placozoa*, one of the earliest multicellular organisms, but remains absent in choanoflagellates [5,9]. At this evolutionary stage, the MDM ancestor does not yet possess E3 ligase activity and appears to regulate the p53 family solely through direct protein-protein complexation. During the evolution of bony vertebrates, concurrent with the distinct genetic divergence of the p53 family, MDM4 (MDMX) emerges as a result of gene duplication [9]. Simultaneously, MDM2 acquires its catalytic ligase activity. Together with MDM4, which retains the ancient, ligase-independent binding mechanism, MDM2 assumes a central role in regulating the p53 family [9].

Around this same period, the ancestor of the Bcl-2 protein family also emerges in *Placozoa* [12,13]. The Bcl-2 family is an evolutionarily conserved group of regulators that govern the mitochondrial pathway of apoptosis. From the perspective of molecular biology and medicinal chemistry, it is particularly noteworthy that both p53 and Nutlin-3, a small-molecule mimetic of the p53 transactivation domain (p53TAD), exhibit the structural capacity to bind not only the MDM proteins but also domains within the Bcl-2 family [14,15,16,17]. Naturally, the Bcl-2 family serves here as a representative example, given its prominence in structural and functional research.

The simultaneous evolutionary emergence of the MDM and Bcl-2 families, both equipped with the ability to interface with the p53 family, indicates that the stringent regulation of cell death and genomic integrity became an indispensable requirement for the evolution of multicellularity. The advent of new regulatory modules, such as the Bcl-2 family, exploited signaling routes that passed through the already established p53 cellular hub. Thus, even at this early evolutionary juncture, the centralization of diverse signaling and effector processes onto the p53 molecule had already begun. Before exploring the hierarchical organization of these processes, a fundamental question must be addressed: just how extensive is the topological scale of the p53 network compared to other cellular proteins?

## 3. p53 Network Scale

To understand the scale of this network, we conducted a brief topological analysis of 15 proteins. These specific proteins were purposefully selected to provide a representative cross-section of diverse fundamental cellular functions—ranging from basic metabolism and cytoskeletal scaffolding to complex immune and apoptotic signaling—distributed across three distinct evolutionary tiers:

1. The Ancients: These proteins are fundamental to life and emerged over 1.5 billion years ago. This group includes Ubiquitin C (UBC; protein degradation tagging), Actin Beta (ACTB; cytoskeleton and motility), Glyceraldehyde-3-phosphate dehydrogenase (GAPDH; glycolysis), Heat Shock Protein 90 (HSP90AA1; chaperone for proper protein folding), and Proliferating Cell Nuclear Antigen (PCNA; DNA polymerase sliding clamp for replication).
2. Multicellular Architects (600–800 Myr ago): This group comprises Proto-oncogene c-Myc (MYC; transcriptional regulation), Beta-catenin (CTNNB1; cell adhesion), Epidermal Growth Factor Receptor (EGFR; growth factor signaling), and Caspase-3 (CASP3; apoptosis execution).
3. Vertebrate Innovations (400–500 Myr ago): This group features Hemoglobin Beta (HBB; oxygen transport), Albumin (ALB; osmotic pressure and molecular transport), Interferon Gamma (IFNG; adaptive immunity), Insulin (INS; glucose regulation), and Prolactin (PRL; lactation and osmoregulation).

**Figure 2:**
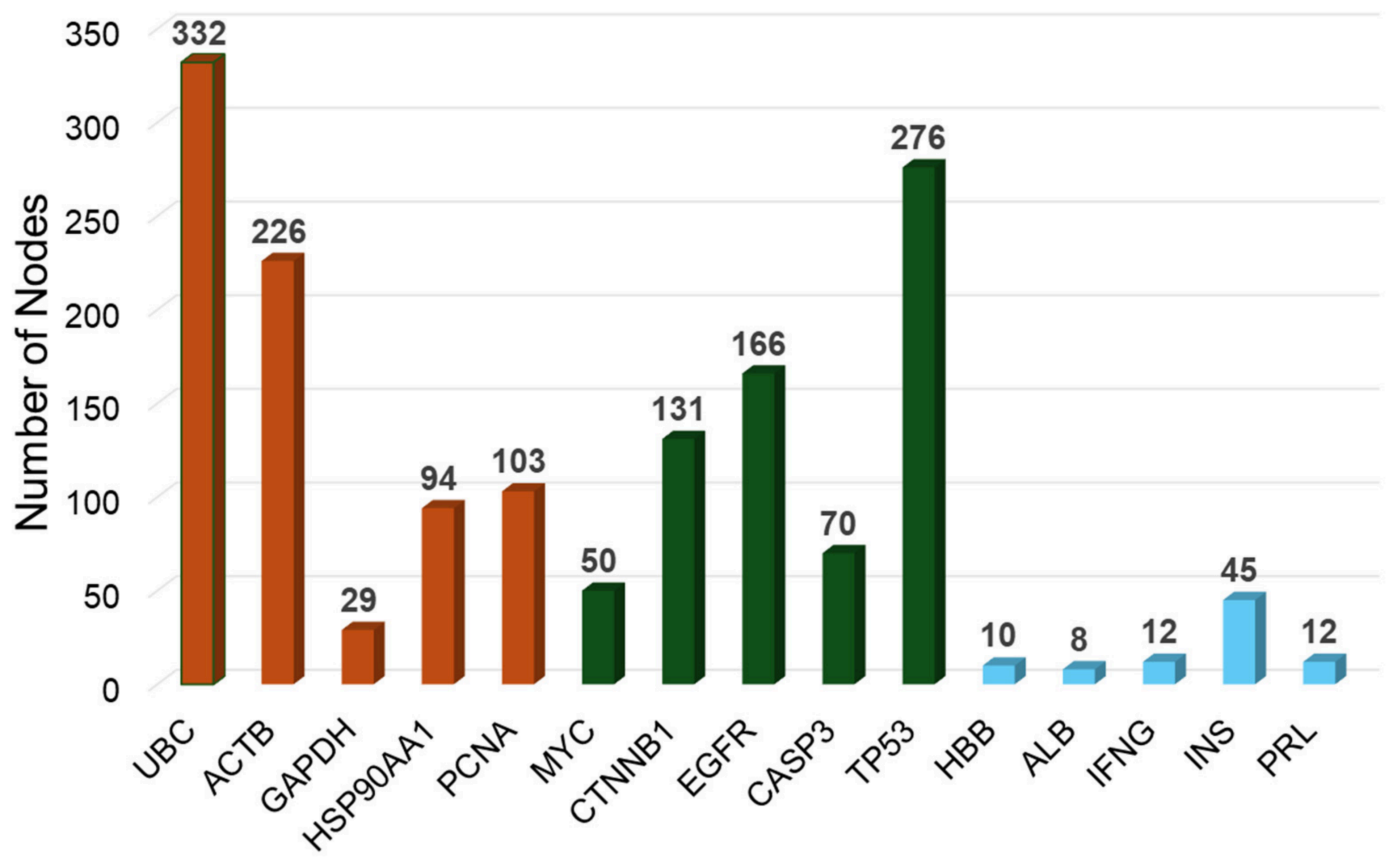
Comparison of interactome complexity across 15 selected proteins of varying evolutionary ages. Interaction data was retrieved from the STRING database (version 12.0), restricted to experimentally validated interactions and curated databases with a high confidence score (>0.700; textmining disabled to prevent literature bias). The proteins are categorized into three evolutionary cohorts: The Ancients (orange), Multicellular Architects (dark green), and Vertebrate Innovations (light blue). Notably, p53 (276 nodes) exhibits a topological connectivity comparable to fundamental ancient proteins such as UBC (332 nodes) and ACTB (226 nodes), far exceeding the network complexity of other proteins within its own evolutionary cohort and more recent vertebrate innovations.

While filtering interaction networks significantly mitigates direct publication-volume bias, it is important to acknowledge that inherent database ascertainment bias remains, as disease-associated and historically older proteins are naturally more intensively investigated in experimental settings. Nevertheless, establishing strict filtering criteria allows for a more standardized structural comparison of topological complexity across proteins of different evolutionary ages (Figure 2). Detailed search parameters and network settings for the STRING database analysis are provided in Section 14.

Because multiple protein–protein interaction form complex networks, they are generally subject to the “rich-get-richer” principle of preferential attachment [18]. According to this rule, the oldest nodes in a network, those already possessing established links, acquire a disproportionately larger number of new connections as the network scales. Within the “Ancients” group, two proteins possess over 200 connections, with UBC reaching a sample peak of 332 nodes. The “Multicellular Architects” group also displays substantial connectivity.

p53, however, presents a pronounced topological anomaly within the multicellular cohort. Despite its significantly later evolutionary emergence, it has assembled a network of 276 nodes, surpassing the scale of most ancient genes and vastly exceeding the connectivity of its evolutionary peers, such as MYC (50) and CASP3 (70). This indicates strong selective pressure that ultimately integrated p53 as the central hub for coordinating cellular stress responses.

Consequently, a profound conclusion emerges: the signaling pathways governing genome integrity, which originated during the transition from unicellular to multicellular life, rapidly became as indispensable as the most ancient fundamental cellular mechanisms. This realization necessitates addressing the following structural question: how is the regulation of such highly centralized networks, characterized by multiple input and output parameters, actually organized?

## 4. Systems Architecture and Hierarchical Organization of p53 Regulation

**Figure 3:**
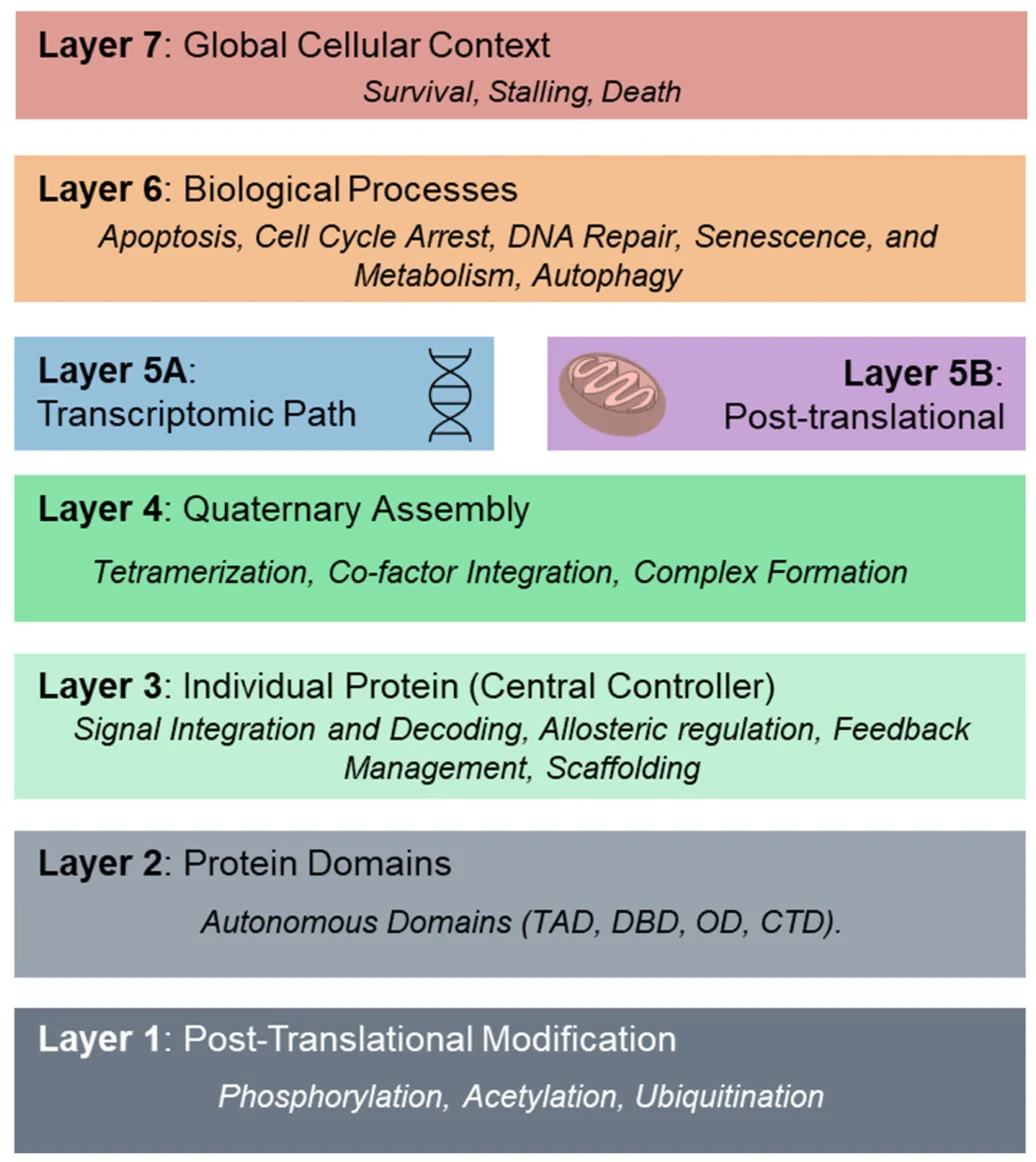
Hierarchical System Architecture of p53-mediated Cellular Control. The model illustrates the flow of biological information from fundamental chemical bits to global cellular fates. Layers 1 and 2 represent the molecular hardware and input code, including post-translational modifications (PTMs) and autonomous functional regions: the transactivation domain (TAD), DNA-binding domain (DBD), oligomerization domain (OD), and C-terminal domain (CTD). Layer 3 acts as the central processing unit (CPU/SoC) for signal integration and decoding. Layer 4 signifies the assembly of the functional unit via quaternary assembly (tetramerization). Layer 5 demonstrates parallel execution through genomic/transcriptomic (5A) and non-genomic/post-translational (5B) pathways, which activate specific biological programs (Layer 6) to shift the cell into one of the global attractor states (Layer 7).

To understand the complexity of the p53-MDM2 interaction and the challenges of its pharmacological modulation, it is essential to define the hierarchical architecture of the p53 network. We propose a multi-layered systems framework (Figure 3) that conceptualizes p53 not merely as a single protein, but as a central information-processing hub or a ‘System-on-a-Chip’ (SoC). This hierarchy spans from the fundamental chemical ‘bits’ of post-translational modifications (Layer 1) to the global attractor states of the cell (Layer 7). By establishing this framework, we can better analyze the selective routing of signals through parallel genomic and non-genomic pathways and identify the structural vulnerabilities that lead to systemic failure in cancer and therapy (Figure 3).

### 4.1. Layer 1: Amino Acids and Post-Translational Modification

At the most fundamental level of the p53 hierarchy (Layer 1), information is encoded through a vast array of site-specific chemical modifications. This level functions as a “chemical reaction layer” where the primary sequence of amino acid residues serves as a substrate for various enzymes. Critically, while the accessibility of these residues is determined by the protein’s fold, the chemical modification itself represents a discrete signal that is relatively independent of the higher-order (tertiary or quaternary) structure at the moment of the reaction. These modifications act as molecular “bits” that decouple external stress sensors from the central decision-making core.

The hallmark of Layer 1 is its extreme variability, both in the types of modifications and the specific residues targeted. A single lysine residue, for instance, can be a site for competitive acetylation, ubiquitination, or methylation, with each modification leading to a fundamentally different systemic outcome. This combinatorial logic allows p53 to integrate a multitude of signals—from DNA damage and oxidative stress to metabolic shifts—into a unified regulatory language [19,20,21,22,23] (Table 1).

### 4.2. Layer 2: Structural Domains (The Functional Interface)

At the second level of our hierarchy, individual amino acid residues are no longer viewed in isolation but are organized into structural domains. A domain represents a discrete unit of tertiary structure—formed by the precise folding of α-helices and β-sheets—that can function relatively independently [34,35,36]. In the context of the p53 SoC model, these domains act as specialized functional ports. These “ports” can be categorized based on their systemic role:

1. Service (Internal) Interfaces: These domains are responsible for the protein’s self-maintenance and structural integrity. For instance, the Oligomerization Domain (OD) is a “service port” dedicated to the critical process of assembly (Layer 4).
2. Primary Functional (External) Interfaces: These domains execute the core instructions of the cell. The DNA-Binding Domain (DBD) is the primary “output port” that interacts with the genome, while the Transactivation Domain (TAD) serves as the “input port” for recruitment of co-factors.

At this level, the spatial geometry of the protein becomes critical. While Layer 1 handles chemical signals, Layer 2 is defined by its structural topology. Consequently, mutations at this level are not merely “data errors” but represent hardware failures. A single “hotspot” mutation in the DBD can physically deform the reading head of the controller, rendering the entire system incapable of executing its tumor-suppressive program, regardless of the signals received at Layer 1 (Table 2).

It is worth noting that in experimental structural biology and pharmacology, the characterization of Layer 2 often relies on the isolation of individual domains. For instance, screening for MDM2/MDMX inhibitors (such as Nutlins [42]) typically utilizes only the isolated Transactivation Domain (TAD) peptide, while thermodynamic stability studies of oncogenic mutants focus exclusively on the core DNA-Binding Domain (DBD) [39]. In systems engineering terms, this reductionist approach is equivalent to Unit Testing—isolating a specific hardware module on a test bench to measure its parameters without peripheral noise. While invaluable for high-resolution structural mapping, this approach possesses a critical limitation: it completely strips the protein of its inter-domain allosteric context. To understand how these isolated ‘ports’ cooperate and compute signals in vivo, we must transition to Layer 3, where the individual domains merge into an integrated, holistic controller.

However, a critical distinction must be made regarding the scale of the pharmacological agent. For classic small-molecule inhibitors designed as orthosteric competitive inhibitors—such as Nutlins—the loss of whole-protein allosteric context is less detrimental. These molecules act as localized structural ‘plugs’ that occupy a discrete hydrophobic pocket within a single isolated domain (Layer 2). For this specific modality, the reductionist ‘unit testing’ approach remains reasonably predictive because the drug’s mechanism of action is mostly independent of the global system state.

Conversely, this model completely breaks down when addressing macromolecular protein–protein interaction (PPIs) or allosteric rescue frameworks (such as mutant p53 reactivators like APR-246) [43,44]. In these scenarios, the therapeutic outcome depends entirely on modifying the long-range conformational dynamics and restoring the internal ‘communication bus’ across the entire monomer. For holistic network interventions, isolating the domain strips the system of the very emergent properties (Layer 3) required for the drug to function.

### 4.3. Layer 3: Individual Protein: Inter-Domain Cooperation and Signaling Architecture

#### 4.3.1. Inter-Domain Cooperation

While Layer 2 defines the structural blueprint of individual domains, Layer 3 represents the emergence of the integrated controller. At this level, p53 ceases to function as a summary of isolated modules and operates as a unified information transducer. The defining feature of Layer 3 is the conformational crosstalk and mutual influence between domains, where a biochemical event at one “port” (such as a post-translational modification at Layer 1) alters the physical properties and affinity of a distant “port”.

This inter-domain communication is fundamentally allosteric and relies on both structured bridges and the flexibility of intrinsically disordered regions (IDRs). Instead of analyzing complex molecular dynamics trajectories directly, the robustness of this internal communication network can be map-validated through a substantial body of experimental literature. These studies confirm that the physical interaction between functionally distinct regions of the monomer forms a coordinated “data bus,” ensuring that input signaling (TAD/CTD) is directly coupled to the primary functional output (DBD) (Table 3).

#### 4.3.2. Signaling Architecture and Information Processing

Having established the interactive architecture among the various autonomous domains, we can now delineate the mechanisms of information processing. At Layer 3, sensor domains (signal-receiving) relay information to effector domains (signal-transducing) through dynamic allosteric interactions. Consequently, the p53 protein functions as a highly sophisticated molecular transducer. A recurring thesis in this review is that the deeper and more extensively a protein is integrated into a systemic informational network, the more formidable it becomes to externally manipulate or pharmacologically control.

**Figure 4:**
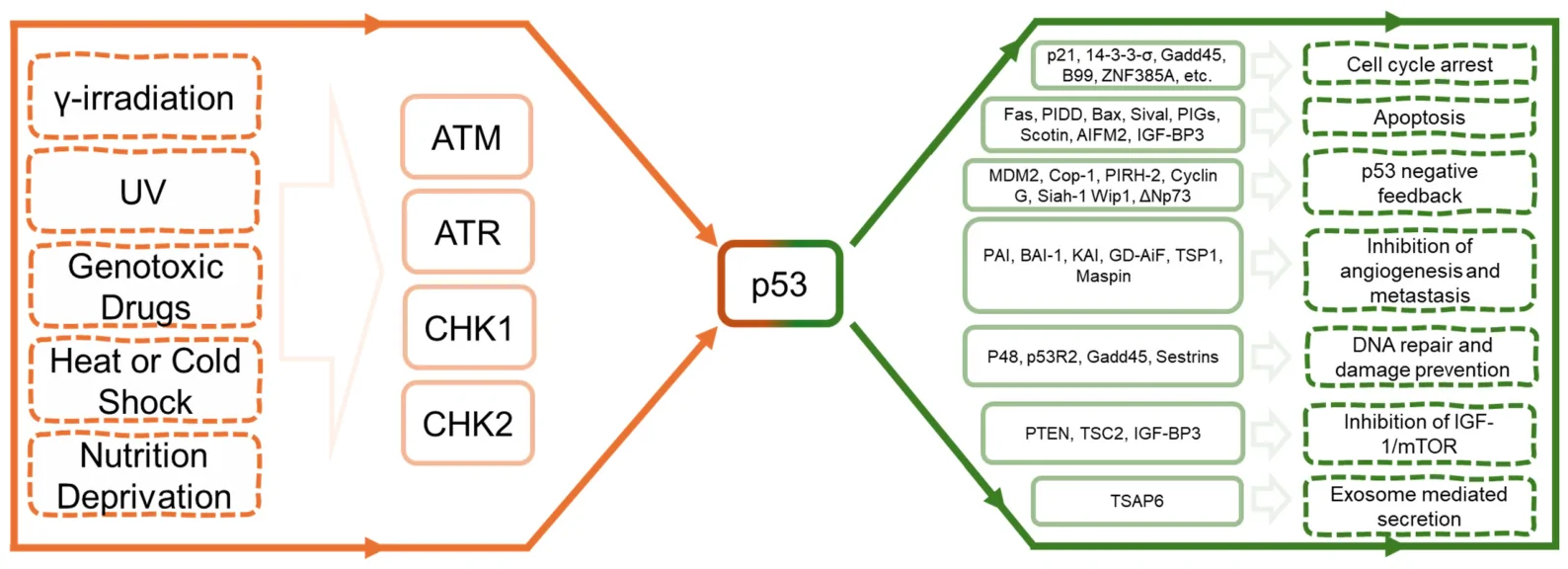
The “bow-tie” network architecture of p53 signaling. The model illustrates the signal compression (“fan-in”) of diverse upstream stress stimuli (orange, left) into the universal p53 central processing hub. Following data integration and decision-making, the network undergoes expansion (“fan-out”) to execute specific, distributed downstream effector programs (green, right). Conceptual schematic independently redrawn and adapted based on the reference pathway architecture from the KEGG database (Pathway: hsa04115, p53 signaling pathway).

In this context, it is crucial to invoke the “bow-tie” biological network architecture formalized by Kitano [52]. This structural framework enables the funneling of massive, diverse incoming stimuli—such as myriad cellular stress signals—into a central, highly conserved core node (p53), which processes the data and subsequently diverges new outgoing signals to execute specific cell-fate decisions (Figure 4). If a direct, independent pathway existed for each of the hundreds of distinct incoming stress stimuli (signal-in) to trigger a corresponding cellular response (signal-out), the system’s complexity would scale exponentially, rendering it energetically untenable, unmanageable, and highly vulnerable to stochastic noise.

Analogous to data transmission protocols in computer science—where the TCP/IP layer bridges a multitude of applications with diverse physical networking media—or trophic webs in ecology, this bow-tie structure provides a robust evolutionary mechanism for “compressing” heterogeneous input information into a universal, multi-functional processing node.

The principal advantage of this bow-tie architecture is signal integration. Rather than acting as a simple biological relay, the central p53 node functions as a dynamic integrator that consolidates diverse incoming perturbations (e.g., DNA damage, oncogenic stress, and hypoxia). Downstream effector responses are triggered only if the cumulative integrated signal exceeds a critical activation threshold. This mechanism effectively filters out stochastic noise, preventing the false activation of drastic cellular defense programs during minor, non-threatening deviations in homeostasis [53].

Within this regulatory context, the p53 network is best visualized as two broad cones converging at a single critical bottleneck (Figure 4). The input cone (“fan-in”) comprises a diverse array of upstream sensors (such as ATM, ATR, CHK1, and CHK2) that detect specific types of cellular damage. The architectural core is p53 itself, which evaluates and decodes the intensity, duration, and combination of these incoming signals.

However, signal decoding at the level of a single protein controller (Layer 3) is biologically insufficient to execute the broad “fan-out” transcriptomic programs required for cell-fate decisions. To translate these decoded signals into a coordinated systemic response and achieve high-affinity binding to genomic targets, the p53 architecture must physically scale up. This structural manifestation of the decoding process dictates the transition to Layer 4: Quaternary Assembly, where individual p53 monomers cooperatively tetramerize to form the fully active functional unit.

### 4.4. Layer 4: Quaternary Assembly

The architectural logic of the signaling network relies on hierarchical integration: cooperative interactions among amino acid residues fold into stable, isolated domains, while the ordered spatial coordination of these domains yields a functionally active monomer. The subsequent systemic step is the physical assembly of these protein subunits. In complex systems engineering theory, this approach is equivalent to the concept of stepwise encapsulation, where the ascent from primary structural components to autonomous functional modules culminates in their integration into distributed multiprocessor clusters [54].

A defining feature of Layer 4 is the fundamental shift in physicochemical bonding modes. While the intramolecular integration of domains (Layers 2 and 3) is driven by robust covalent bonds and internal structural rearrangements, interactions at this quaternary level bifurcate into two distinct pathways.

On one hand, regulation is mediated indirectly through cascades of chemical reactions (e.g., the enzymatic phosphorylation of downstream proteins). On the other hand, it is executed via direct intermolecular contacts, which serve as the primary focus of analysis at this layer. This type of coordination encompasses both heterotypic interactions between p53 and external regulators—such as MDM2, MDMX, and specific DNA binding sites—as well as homotypic oligomerization, namely the assembly of identical p53 monomers into a single functional tetramer [55].

Direct contacts fulfill a dual purpose. They not only induce spatial conformational changes within the proteins themselves or their binding partners, but they also strictly dictate the macroparameters of the entire network—specifically, the pool of thermodynamically free, biologically active protein. For example, the interaction between p53 and its primary negative regulator, MDM2, acts as a dynamic buffer: the binding of monomers temporarily sequesters p53, effectively dampening stochastic input noise and balancing the incoming load to the system [56,57].

The process of tetramerization—the convergence of four p53 monomers into a singular complex—holds particular significance at this level. Evolution co-opted this mechanism as a structural superstructure for precise, non-linear control. As will be demonstrated later, the dissociation constant of the tetramer establishes a critical concentration gradient that heavily influences the transition of p53 from a continuous analog sensor toward a discrete digital switch [58].

**Figure 5:**
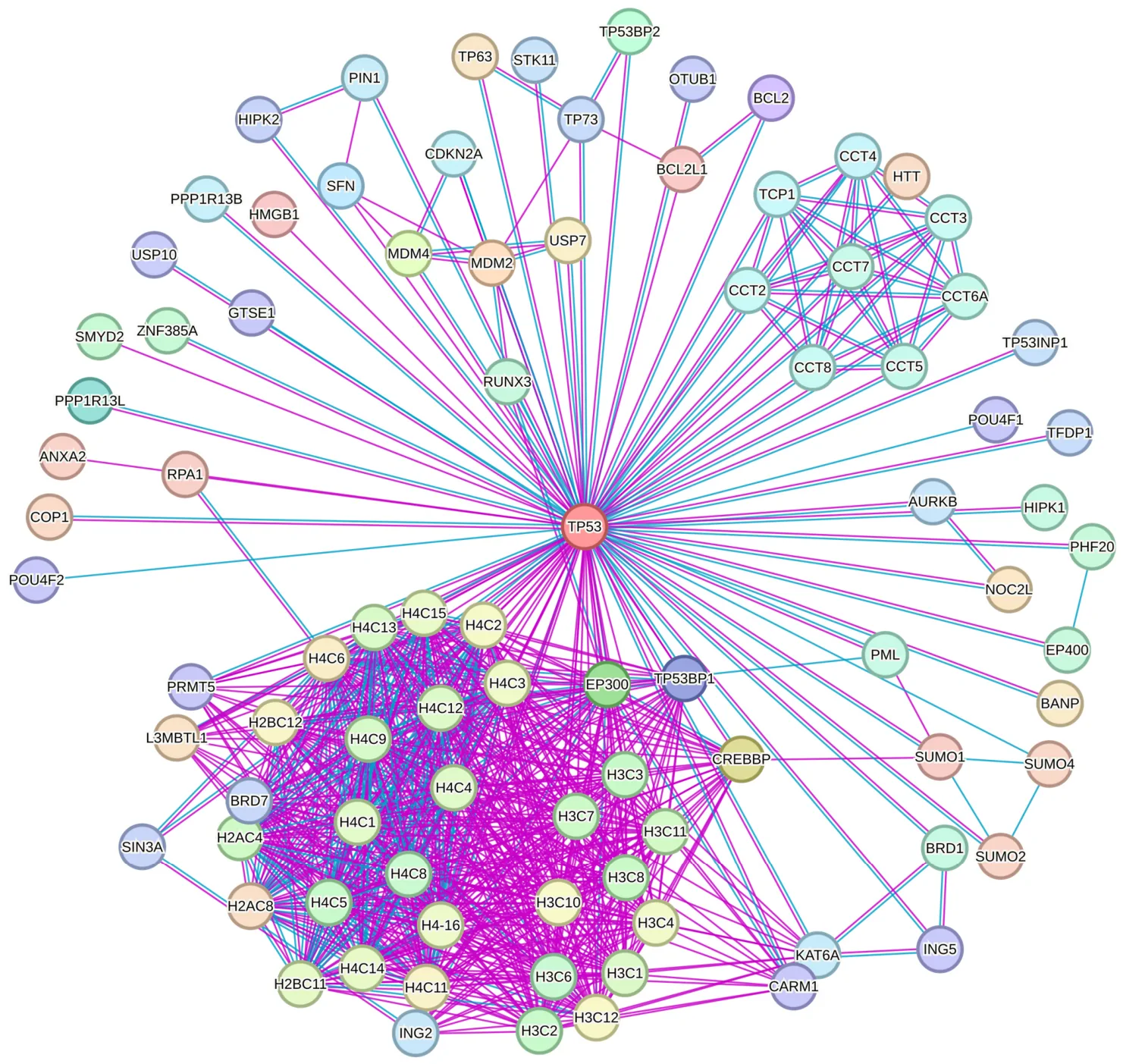
Topological mapping of the p53 interactome as a central cellular super-hub. Generated via the STRING database (version 12.0; Homo sapiens; query date: January 2026; confidence threshold > 0.700), the network graph illustrates the ultra-dense local clustering surrounding TP53. The protein functions as a universal molecular switchboard, leveraging the structural plasticity of its quaternary interfaces to simultaneously integrate massive upstream signals and route control vectors to diverse, independent downstream subsystems (including MDM2/MDMX regulatory loops, epigenetic modulators, and effector cascades).

The massive volume and high density of these connections transform p53 into a super-hub coordinating a distributed network of concurrent cellular processes. The protein’s molecular interfaces possess high plasticity, allowing it to simultaneously serve as an assembly point for hundreds of signaling pathways. This structural complexity is vividly illustrated in the p53 interactome map retrieved from the STRING database (Figure 5). The graph reveals the formation of an ultra-dense local cluster where p53 acts as the central switchboard, routing control vectors to independent downstream cellular subsystems [59].

### 4.5. Layer 5: Methods of Information Transmission by the p53 Protein

Following the structural assembly of the functional tetramer at Layer 4, the architectural framework progresses to Layer 5, where the central controller physically routes the decoded signal to executioner subsystems through parallel genomic and non-genomic pathways.

#### 4.5.1. Layer 5A: The Transcriptomic Pathway

**Figure 6:**
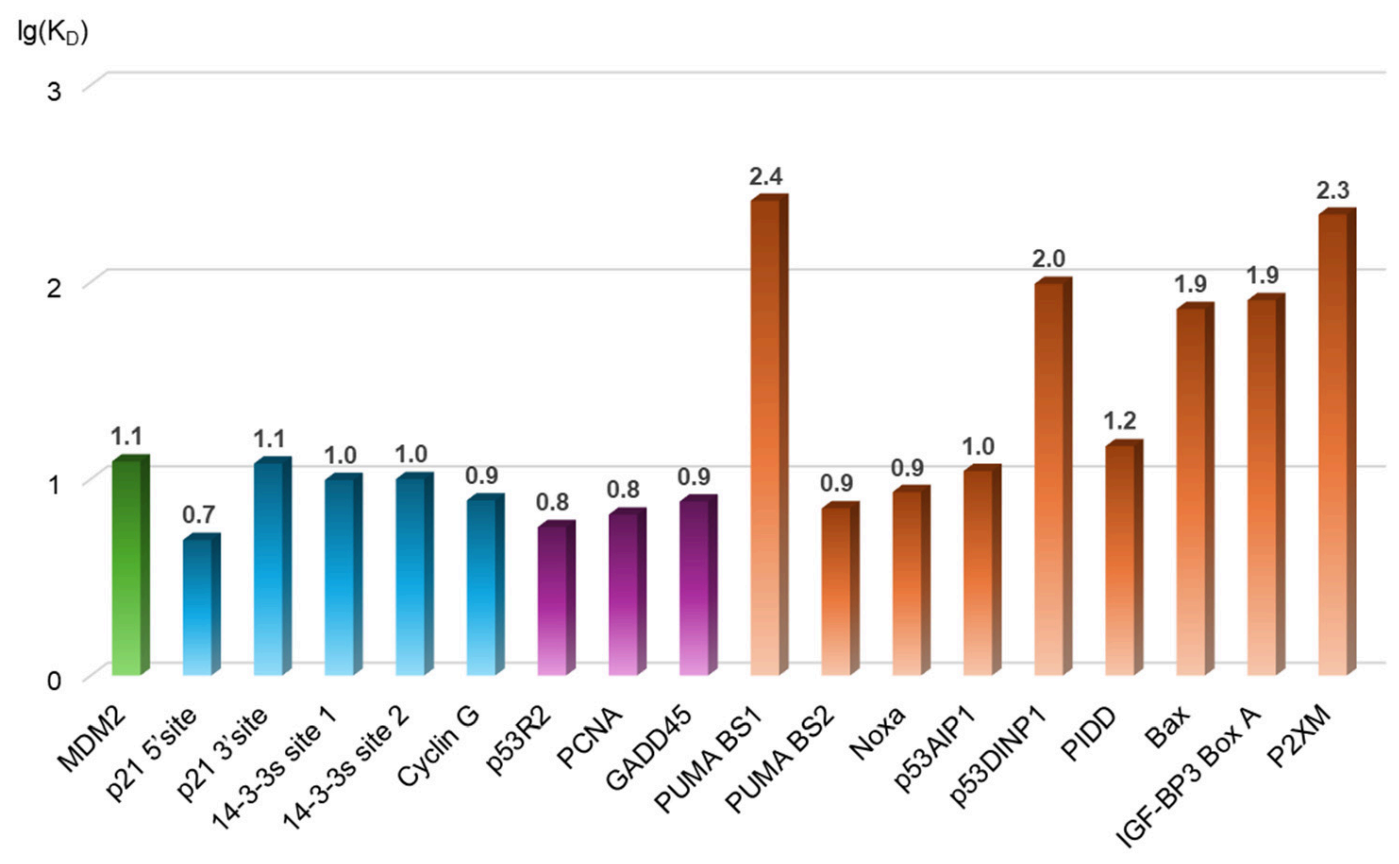
Thermodynamic affinity gradient of p53 for various target gene promoters. The chart displays the base-10 logarithm of the dissociation constant (*lg*(*K*_d_)) for different p53 response elements. Promoters governing cell cycle arrest, DNA repair, and negative feedback loops (e.g., *MDM2*, *p21*, *GADD45*) exhibit lower *K*_d_ values (higher thermodynamic affinity), ensuring their prioritized activation at low, basal p53 concentrations. Conversely, promoters associated with apoptosis (e.g., *BAX*, *PUMA*, *PIDD*) possess significantly higher *K*_d_ values (lower affinity), requiring a massive accumulation of pre-assembled p53 tetramers to surpass the functional activation threshold.

The differential activation of cellular programs at the transcriptional level is dictated by the heterogeneous binding affinities of p53 to the promoters of its target genes. Studies by Alan Fersht’s group have demonstrated that the promoters of genes responsible for cell cycle arrest (such as *p21*) and DNA repair exhibit high thermodynamic affinity for p53, ensuring their prioritized expression even at minimal protein concentrations [55]. In contrast, key apoptotic effectors contain low-affinity recognition sites. For instance, p53 binding to the *BAX* promoter requires a protein concentration that is, on average, an order of magnitude higher [60,61] (Figure 6, Table 4).

This biochemical barrier is substantially reinforced by the regulator’s tetramerization mechanism. The p53 protein functions predominantly as a tetramer (a dimer of dimers), existing in the nucleoplasm in a state of dynamic dimer-tetramer equilibrium. Under physiological conditions (37 °C), the dissociation constant of the tetramer into dimers (*K*_d(*T*−*D*)_) is approximately 50–150 nM [58]; consequently, under basal conditions, the protein pool consists predominantly of largely inactive dimers.

Concurrently, the interaction of p53 with DNA is highly cooperative. For high-affinity promoters, the cooperative assembly of a stable protein-DNA complex is possible even at low nuclear p53 levels, as these specific sequences act as molecular templates, stabilizing the protein and inducing in situ tetramer assembly directly on the regulatory element [46,55]. However, targeting the low-affinity sites of apoptotic genes requires overcoming a critical concentration threshold (above 150 nM). Surpassing this barrier shifts the solution equilibrium toward the formation of pre-assembled, functionally active tetramers. The binding of such a fully formed tetramer to DNA proceeds via a “double-clamp” mechanism, which amplifies the binding affinity to the target locus by more than 50-fold [62].

The divergence in dissociation constants (*K*_d_), combined with the stringent requirements for cooperative tetramer assembly, maximizes system reliability. This structural failsafe prevents the accidental triggering of lethal apoptotic programs unless the severity of the stress induces a massive accumulation of p53 in the nucleus.

It is crucial to emphasize, however, that while this affinity-driven routing provides an attractive biophysical framework, cell-fate selection in vivo cannot be reduced solely to p53 concentration and promoter *K*_d_. The actual transcriptional output represents a highly integrated response. In the complex nuclear environment, p53 promoter selectivity is profoundly modulated by chromatin accessibility, specific response-element architecture, and the recruitment of tissue-specific cofactors. Furthermore, the precise combination of post-translational modifications (Layer 1), p53 temporal pulse dynamics (Layer 5C), metabolic status, cell lineage, and cooperative interactions with other transcription factors collectively dictate the final biological outcome. Thus, while the concentration-dependent tetramerization threshold acts as a fundamental structural failsafe, it operates within a highly nuanced, multidimensional cellular context [63].

#### 4.5.2. Layer 5B: The Post-Translational Pathway

Beyond transcriptional selectivity at the promoter level, post-translational regulation at the mitochondria makes a critical contribution to cell-fate determination. This architecture can be described as a two-layer cascaded signal filtration system.

The first layer executes regulation at the signal “input”: the E3 ubiquitin ligase MDM2 directly controls the global p53 concentration via proteasomal degradation, maintaining it at a basal level in the absence of stress. The second filtration layer is deployed at the signal “output” of the system and is controlled by the Bcl-2 protein family on the outer mitochondrial membrane. Even if p53 successfully breaches the first barrier and transcriptionally activates the pro-apoptotic effector BAX, the execution of the death program is not instantaneous. Anti-apoptotic proteins (such as Bcl-2 and Bcl-xL) function as a molecular and stoichiometric buffer: they physically sequester active BAX molecules, preventing their oligomerization and the subsequent permeabilization of the mitochondrial membrane.

Consequently, Bcl-2 proteins establish an autonomous resistance threshold within the network. Under physiological conditions, the capacity of this buffer is sufficient to neutralize minor BAX fluctuations caused by background noise or weak, repairable genomic damage. The system’s transition into the apoptotic state becomes possible under two scenarios. The first is the classical accumulation of extremely high concentrations of active p53, wherein the pool of synthesized BAX completely overloads and exhausts the Bcl-2 buffering capacity [64]. The second is the pharmacological inhibition of Bcl-2 itself (for example, using BH3 mimetics).

As demonstrated by Kracikova et al., at isolated low levels of p53, the cell predominantly selects the temporary cell cycle arrest program [65]. However, the combined application of low-dose p53 activators and Bcl-2 inhibitors drastically lowers the apoptotic transition threshold [65]. In systems engineering terms, this synergistic effect operates as a catalytic reduction in activation energy: the drug combination does not increase the physical magnitude of the incoming p53 signal, but rather topologically lowers the resistance barrier of the downstream mitochondrial interface, forcing the system to switch into the death attractor even under weak fluctuations of the regulator.

**Figure 7:**
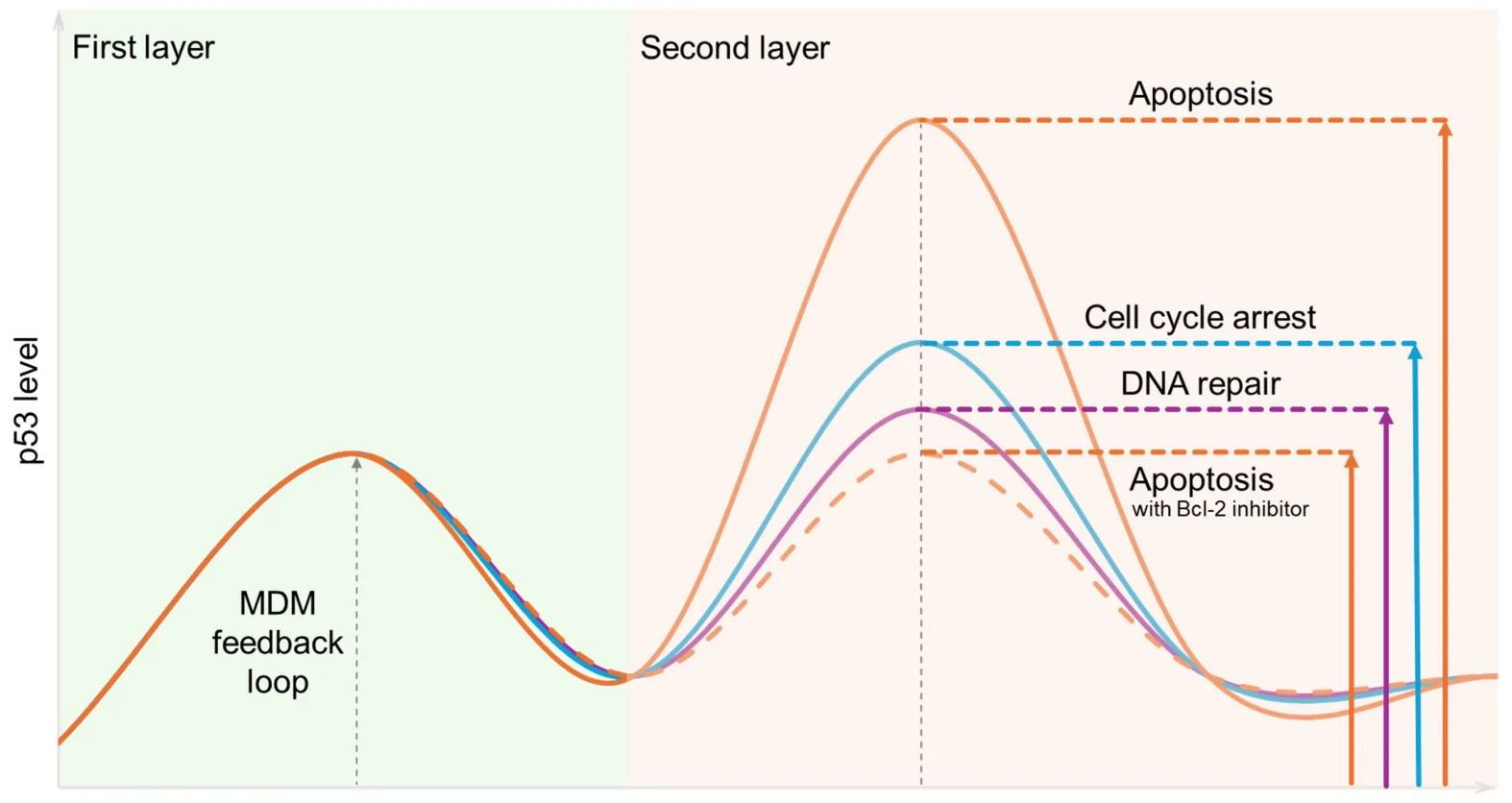
The two-layer cascaded filtration of the p53 signal and concentration-dependent cell-fate determination. The first layer (green shading) represents the primary regulatory threshold at the signal input, governed by the MDM-p53 negative feedback loop, which dampens basal fluctuations and noise. The second layer (orange shading) illustrates the downstream signal output governed by target gene affinity and mitochondrial Bcl-2 buffering. While transient or moderate p53 levels selectively trigger DNA repair and cell cycle arrest, only a massive, sustained accumulation can breach the high threshold required for apoptosis (solid orange curve). Notably, the pharmacological blockade of the Bcl-2 buffer (dashed orange curve) catalytically lowers this resistance barrier, enabling the system to transition into the apoptotic attractor at significantly lower p53 concentrations.

The architecture described above guarantees high response fidelity: random noise is dampened at the promoter level (Layer 5A) or neutralized by downstream buffering proteins (Layer 5B), whereas a genuine, sustained stress signal successfully overcomes both barriers (Figure 7).

#### 4.5.3. Layer 5C: Temporal Signal Modulation (Oscillatory Dynamics)

**Figure 8:**
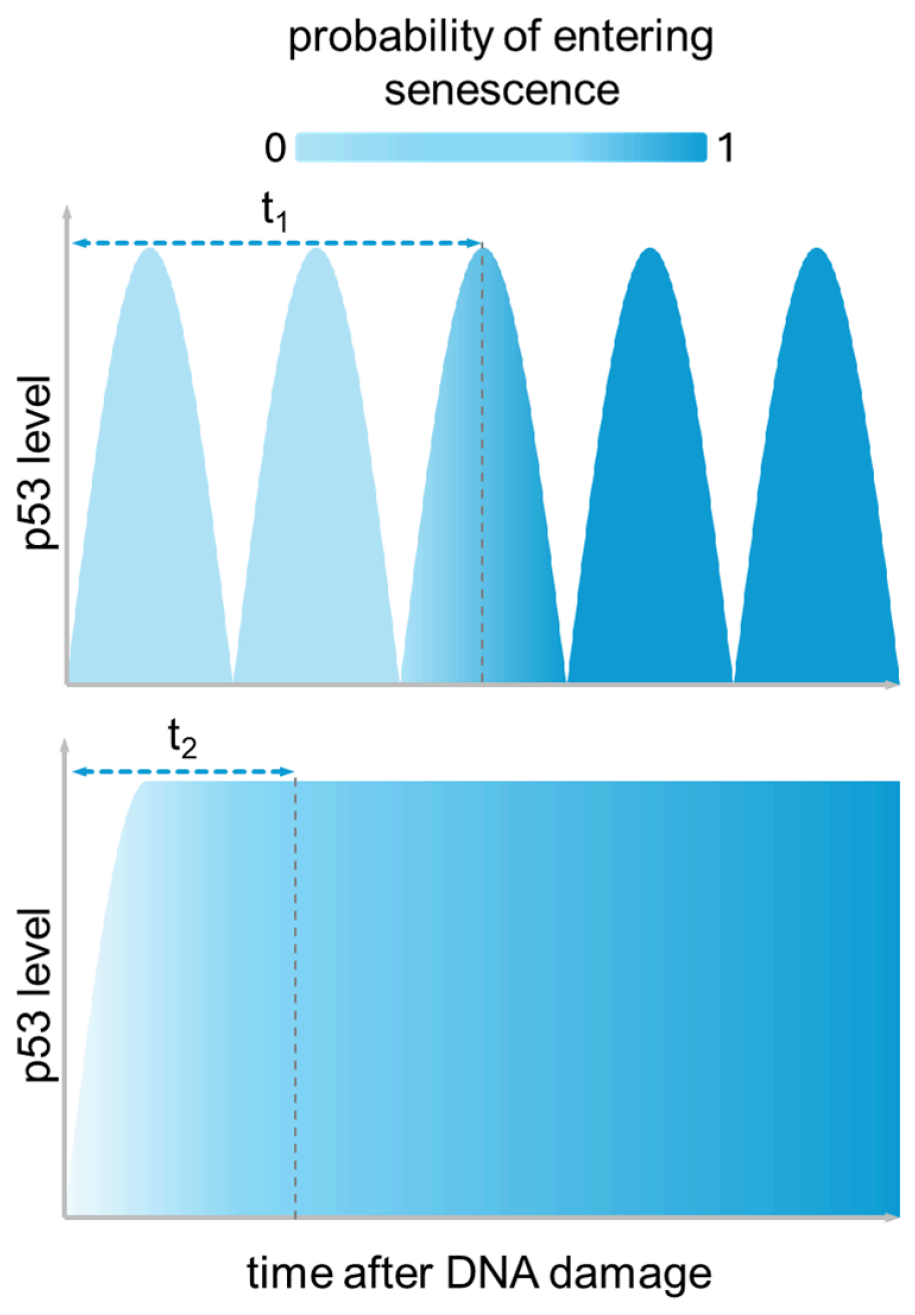
Temporal encoding of cell fate via p53 oscillatory dynamics [69]. The upper panel illustrates the physiological pulsatile mode (digital encoding), where discrete oscillations of p53 provide the cell with a prolonged “decision-making window” (*t*_1_) to attempt DNA repair. In this mode, the probability of entering senescence increases gradually and is strictly dependent on the cumulative number of cycles. The lower panel depicts a sustained, static p53 activation (analog encoding), which forces an accelerated, irreversible transition into senescence or apoptosis (*t*_2_ < *t*_1_). This temporal modulation acts as a crucial dynamic filter, preventing premature terminal responses to transient stressors.

The functional architecture of the p53 network is realized not within a static coordinate system, but through complex temporal patterns [66]. Under physiological conditions, the response to genotoxic stress does not manifest as a monotonic plateau of protein concentration, but rather as a series of discrete pulses (oscillations) with a fixed period and amplitude [67]. This stable rhythm is generated by a temporal delay within the negative feedback loop formed by the p53–MDM2 axis [68] (Figure 8).

From a control theory perspective, this transition from analog (continuous) to digital (discrete pulse) signal encoding fundamentally alters the robustness of the entire system. Static hyperactivation of the hub—characteristic of crude pharmacological intervention or terminal cellular damage—forcibly drives the system into a continuous plateau, triggering an irreversible and chaotic cascade of death signals. Conversely, the pulsatile operational mode preserves the system’s ability to safely revert to its basal homeostatic state, provided the stressor is resolved [69].

Oscillatory dynamics enable the cell to execute genome monitoring with active feedback. The temporal lag between the peak of p53 accumulation and the subsequent transcriptional response of MDM2 creates a dynamic “decision-making window,” during which the system evaluates the progress and success of DNA repair processes. As demonstrated in temporal dynamics studies, the ultimate selection of cell fate is encoded not by the peak signal amplitude, but by the cumulative number and duration of the sustained pulses [69].

Transient oscillations confine the cell within the reversible trajectory of DNA repair, whereas exceeding a critical cycle limit signals unresolvable damage, driving the system into the terminal, irreversible attractors of apoptosis or cellular senescence [69]. Thus, frequency-pulse modulation serves as a paramount dynamic filter, safeguarding the distributed cellular network against fatal false-positive activations in response to stochastic homeostatic noise.

### 4.6. Layer 6: Cellular Attractors

The integration of transcriptional affinity constants, cooperative quaternary assembly mechanisms, and frequency-pulse modulation elevates the p53 information network to the next macroscopic level. In this system, discrete cellular states represent stable, multidimensional attractors within the gene expression space, corresponding to Sui Huang’s fundamental concept of potential landscapes [70]. The p53 super-hub itself is not a fixed coordinate on this landscape; rather, it acts as its primary dynamic operator. By modulating its concentration, post-translational code, and temporal signal profile, p53 physically deforms the landscape topology, flattening energy barriers or generating new basins of attraction for the system’s current state [71].

The topography of the signaling network is structured around several basal attractors, which are topologically distributed between two evolutionary macro-strategies defined by Hiroaki Kitano: the preservation of the initial phenotype (“Stay”) or its irreversible alteration (“Change”) [53].

**Figure 9:**
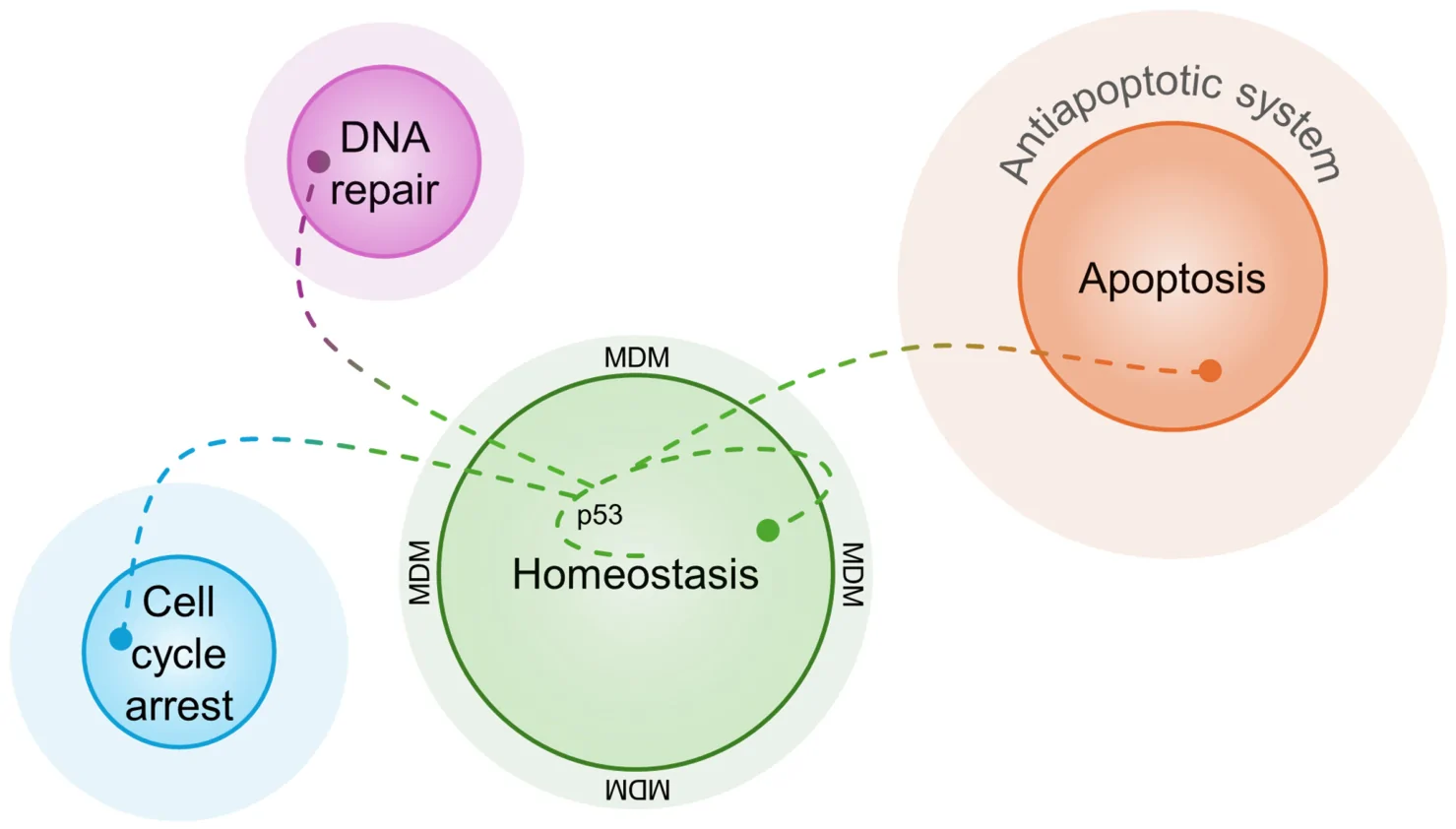
The potential landscape of p53-governed cellular attractors. The network topography is defined by multiple stability basins. Under basal conditions, the cell is anchored in the “Homeostasis” attractor, guarded by the MDM2 negative feedback barrier. Moderate stress shifts the trajectory into reversible “DNA repair” and “Cell cycle arrest” basins (the “Stay” macro-strategy). Overwhelming or sustained stress breaches the threshold barriers (dashed paths), escaping the MDM2 boundary and overcoming the antiapoptotic buffer system to irreversibly fall into the terminal “Apoptosis” attractor (the “Change” macro-strategy).

In the absence of external perturbations, a healthy cell resides in the basal homeostasis attractor (Figure 9). This stable state is characterized by minimal p53 levels, tightly constrained within a local minimum by the stringent negative feedback barrier exerted by MDM2 [71,72].

Upon the onset of moderate genotoxic stress, the cellular trajectory shifts into the attraction basin of temporary cell cycle arrest and DNA repair, marking the initiation of the “Stay” macro-strategy. This state is highly dynamic and fully reversible: the system is maintained within this basin exclusively by the frequency-pulse operational mode of p53 [69,72]. Discrete peaks in protein concentration selectively activate only high-affinity repair and arrest promoters (such as *p21*), enabling the cell to conduct stepwise genomic surveillance, preserve phenotypic stability, and avoid crossing into terminal zones.

If DNA damage proves irreparable and is not resolved within a threshold number of pulses, the system encounters a mathematical bifurcation. p53 exits the pulsatile mode and establishes a stable, analog high-concentration plateau, reinforced by phosphorylation at the Ser46 residue and the activation of a positive feedback loop via PTEN [71,72].

From a systems engineering perspective, this switch operates analogously to an electronic Schmitt trigger exhibiting pronounced hysteresis [73,74]. The high energy barrier (separatrix) that previously divided the reversible and fatal trajectories instantaneously vanishes at the bifurcation point, reconfiguring the landscape geometry of the entire signaling network [71,72]. The presence of hysteresis at this juncture holds critical systemic importance: it effectively suppresses stochastic fluctuations and irreversibly propels the system’s trajectory into the domain of the “Change” macro-strategy.

Here, the system’s trajectory bifurcates, converging toward one of two alternative final attractors. The primary resolution is the apoptosis attractor—the terminal state of programmed cell death. It captures the system when the continuous analog plateau of p53 generates a critical mass of accumulated signals required to execute cell death.

The secondary, alternative resolution is the cellular senescence attractor. The cellular trajectory is redirected into this deep, stable minimum under conditions of prolonged stress if, for instance, the mitochondrial permeabilization threshold for apoptosis is blocked—potentially due to a tissue-specific overexpression of the Bcl-2 buffering capacity [71]. In this state, the cell permanently relinquishes its replicative potential, completely isolating the damaged genome from the broader population while retaining basal metabolic activity.

## 5. Self-Organized Criticality of the p53 Network

Building upon the preceding evidence, a fundamental systemic thesis emerges: the intensity of the p53 signal, expressed through its intracellular concentration, represents not merely a quantitative metric, but a qualitative regulatory code. Regulatory mechanisms at the underlying structural layers translate a linear increase in protein concentration into a discrete, qualitative selection of specific cellular fates. Consequently, the artificial, forced hyperactivation of this hub may not yield a linear amplification of the desired therapeutic outcome; rather, it risks generating informational chaos.

The transition of complex, self-organizing systems from an ordered regime into chaotic dynamics is thoroughly described by the theory of Self-Organized Criticality (SOC) proposed by Per Bak [75], alongside Stuart Kauffman’s concept of the evolutionary self-organization of dynamical systems [76]. Within this theoretical framework, the cascade of p53 output signals operates as a branching process. The defining metric of such processes is the branching ratio (*σ*), which determines the average number of downstream “child” network nodes activated by a single “parent” node during the preceding step [77].

Throughout evolution, many biological systems have been optimized to operate precisely at the critical point of a phase transition—poised on the boundary between rigid order and absolute chaos, where *σ* ≈ 1. It is specifically within this critical state that the network achieves maximum efficiency and bandwidth capacity for information transmission [78,79,80].

However, under conditions of artificial p53 hyperactivation, the branching ratio avalanches, escaping into the supercritical regime of *σ* ≫ 1. At this juncture, the coherent control signal rapidly degenerates into stochastic noise, severely overloading the communication channels. As demonstrated in the preceding section on dynamical attractors, such excessive stimulation strips the system of its capacity to confine its trajectory within the targeted operational basin. Consequently, the cell loses coordination across its functional modules, resulting in a chaotic transcriptional response and erratic switching between mutually exclusive cellular programs.

## 6. Coupling Metrics: The Case of p53

A related approach is examined in software engineering, where a key metric for architectural analysis is Afferent Coupling (*C_a_*)—the number of external components that depend on the operation of a specific module [81,82]. The higher the *C_a_* value, the greater the responsibility placed upon that element, which inevitably renders the system rigid: any modifications to such a central node trigger a cascading “domino effect” throughout the entire structure.

Within the cellular signaling network, the p53 protein exhibits an extreme level of afferent coupling. However, attempting to directly transpose classical software design equations (such as Robert C. Martin’s Instability Metric) into biology introduces a methodological challenge. In IT architecture, connections between modules are strictly defined and discrete, whereas in a living system, these interactions are characterized by varying priorities and thermodynamic strengths. For instance, phosphorylation at one specific amino acid residue may execute a critical command function, while modification at another site may serve only a secondary, modulatory role.

Consequently, the afferent coupling metric for p53 cannot be evaluated merely as an abstract, cumulative sum of input and output signals without accounting for their relative biological weights. Instead, the coupling metric must be defined contextually for each specific downstream biochemical process, namely apoptosis, cell cycle arrest, DNA repair, and metabolism.

From a systems engineering perspective, sustaining extremely high *C_a_* values simultaneously across multiple critical execution programs is direct evidence of a violation of the Single Responsibility Principle (SRP). While the other SOLID design principles are largely upheld within the p53 architecture—primarily due to the strict evolutionary conservation of its autonomous domains and the precise selectivity of its binding interfaces—it is precisely this structural “blurring” of responsibilities among functional modules that introduces a fundamental systemic vulnerability.

Transitioning from a qualitative description of this vulnerability to a quantitative assessment is made possible by utilizing the affinity constants (*K_D_*) presented in Table 4. The order-of-magnitude disparities in p53 binding affinity across different promoters dictate the true biological “weight” of each functional dependency. This framework allows us to mathematically model the intersection density of control vectors at this single point of failure (SPOF) and to pinpoint the threshold where the extreme centralization of p53 ceases to be an evolutionary advantage, becoming instead a destructive liability during attempts at artificial pharmacological regulation.

Nevertheless, even on a qualitative level, this analysis draws a direct parallel between p53 and critical architectural anti-patterns in software design (as detailed in Table 5, which evaluates the p53 network through the lens of the five SOLID principles).

In computer science, this phenomenon is famously termed a “God Object”—a highly centralized module that “knows” or “does” too much. In biological networks, just as in digital ones, such super-hubs inevitably become the ultimate operational bottleneck and the primary point of failure for the entire system.

## 7. p53 from the Perspective of Scale-Free Network Theory

The evolutionary trajectory that transformed p53 into a functional analogue of a “God Object” is entirely logical. From the standpoint of classical software engineering (specifically the Robert C. Martin principles discussed earlier), such extreme centralization appears to be a severe architectural flaw. However, the theory of complex networks elucidates why the living system was compelled to adopt this specific structural path. The emergence of such super-hubs is an inevitable consequence of systemic development governed by the laws of scale-free networks [18].

The primary growth mechanism of these systems is the principle of preferential attachment, commonly known as the “rich-get-richer” rule. According to this model, any novel elements arising during evolution are exponentially more likely to connect to pre-existing major hubs rather than to isolated, peripheral nodes. Because p53 emerged during the earliest stages of metazoan multicellularity, it inherently served as the primary topological anchor for the entire nascent cellular signaling system. Evolutionarily, it was vastly more “cost-effective” and efficient to wire new defensive circuits into an already functional, robust controller than to construct parallel regulatory hubs from scratch [83].

This extreme centralization culminated in the formation of the intracellular “bow-tie” architecture, wherein p53 occupies the absolute bottleneck. The primary function of this topology is to aggregate and integrate a massive influx of diverse stress signals from broad upstream pathways, routing them into a unified central processing protocol. Here, p53 acts as a universal switchboard; the granular contextualization of the incoming data and the ultimate selection of the physiological scenario (cell cycle arrest, DNA repair, or apoptosis) occur further downstream. This downstream resolution is executed either at the level of genomic regulatory element recognition or via the intervention of secondary structural buffers. Consequently, the exceptionally high connectivity of p53 (276 nodes, as demonstrated in our earlier analysis) is the product of historical network inertia rather than the outcome of an intentional, optimized design.

However, it is precisely this network topology that dictates the dual nature of cellular survival. Scale-free biological networks exhibit a fundamental property often formulated as being “robust yet fragile”—highly resilient against random errors and systemic noise, yet profoundly vulnerable to targeted attacks [84,85].

## 8. Mutations as an Attack on Fragility

In the context of oncogenesis, this systemic fragility is exploited with maximum efficiency. Random mutations occurring at the network periphery—within genes characterized by a low degree of connectivity (i.e., low afferent coupling, *C_a_*)—have virtually no impact on global cellular stability. Thanks to high system redundancy and the presence of parallel signal transduction pathways, such localized functional failures are rapidly compensated for, allowing the intact p53-driven genomic surveillance system to promptly eliminate the damaged cell. Consequently, an evolutionary strategy focused on attacking peripheral network nodes proves highly inefficient for tumor development.

The paradigm shifts drastically when a mutation strikes the central hub itself, fundamentally transforming p53 into a Single Point of Failure (SPOF). However, oncogenic selection proceeds with even greater precision, systematically optimizing the topology of this attack. As demonstrated by the work of Arnold Levine and colleagues, the structural strike within the super-hub is directed precisely at its most critical functional module: the DNA-binding domain (DBD) [86]. Approximately 90% of all missense mutations identified in human cancers are clustered within the codons of this specific structural core [86,87,88].

The deactivation or severe structural distortion of the DBD represents a targeted sabotage of the primary, transcriptional pathway of information transmission within the p53 network. Rather than executing a protracted and statistically improbable sequential knockout of hundreds of individual downstream target genes, the tumor lineage is only required to deactivate a single universal reading interface. As a result, the protein completely loses its capacity to recognize genomic regulatory elements, thereby stripping the cell of its deterministic decision-making protocol for self-elimination.

Furthermore, the massive accumulation of this defective hub protein does not merely dismantle the canonical defense system; it frequently confers novel oncogenic properties (Gain-of-Function) [89,90,91,92]. This pathological gain introduces profound informational chaos into residual regulatory cascades and actively accelerates the subsequent evolution of the cancer genome. Thus, the overwhelming dominance of mutations specifically localized within the p53 DBD is the mathematically logical outcome of evolutionary selection driving the fastest and most devastating breach of the cell’s systemic vulnerability.

## 9. Therapeutic Approaches to Targeting the p53 Network

The restoration of p53 function has remained a cornerstone objective in targeted oncology for several decades. Over the years, numerous pharmacological strategies have been developed to re-engage this tumor suppressor network, ranging from small-molecule inhibitors of negative regulators to advanced gene therapies and targeted protein degraders (PROTACs). Extensive overviews of the current therapeutic landscape, evolving drug modalities, and recent clinical trial outcomes have been thoroughly documented in recent comprehensive literature reviews [87,93,94]. Broadly, these interventions can be categorized into distinct conceptual strategies, the most prominent of which historically relies on the direct structural blockade of protein–protein interaction.

### 9.1. The Classical “Lock-And-Key” Therapeutic Approach (Direction 1)

Historically, targeted pharmacology has relied on the “lock-and-key” concept, which serves as the foundation of Structure-Based Drug Design (SBDD). This paradigm, which postulates the geometrically precise insertion of a small molecule inhibitor into a deep active site of a target protein, has yielded monumental clinical triumphs, such as targeted kinase inhibitors. However, within the context of the p53 network, this classical approach encountered a fundamental structural obstacle: the DNA-binding interface of the tumor suppressor itself is an expansive, relatively flat surface devoid of deep hydrophobic pockets. For decades, this topological feature rendered p53 seemingly “undruggable” [95].

Consequently, the vector of pharmacological intervention was redirected toward the hub’s primary negative regulator—the MDM2 protein. Crystallographic studies revealed that MDM2 possesses a deep, well-defined hydrophobic cleft (serving as the “lock”) into which p53 integrates via its alpha-helical transactivation domain, utilizing three critical amino acid residues: Phe19, Trp23, and Leu26 (functioning as the “key”) [96].

The discovery of the small-molecule Nutlin class in 2004 marked a landmark achievement for this paradigm [42]. Nutlins were rationally designed so that their chemical structure spatially mimics the exact topographical arrangement of these three p53 amino acids. By occupying the MDM2 binding pocket, the drug sterically displaces p53 from the complex, thereby preventing its ubiquitination and subsequent proteasomal degradation. Initially, it was anticipated that such an elegant disruption of a localized protein–protein interaction (PPI) would provide a universal strategy for the non-genotoxic reactivation of p53 in tumors harboring wild-type *TP53*.

However, the transition of this elegant structural concept in vivo encountered an insurmountable translational barrier. Success at the localized stereochemical level (Layer 2) culminated in a massive failure at the level of global network dynamics. Firstly, a rigorous distinction must be drawn between the physiological activation of p53 and its forced pharmacological hyperactivation. Under physiological conditions, the p53 regulatory circuit is governed by dynamic feedback loops. During pharmacological intervention (e.g., using small molecules of the Nutlin class), the transcriptional activation of the *MDM2* gene is formally preserved; however, the efficiency of the negative feedback loop drops precipitously. This occurs because the synthetic inhibitor possesses a significantly higher binding affinity for the hydrophobic pocket of MDM2 compared to the natural p53 transactivation domain [97]. Consequently, newly synthesized MDM2 molecules are immediately blocked by the drug, disabling the natural degradation mechanism and isolating p53 from retroactive control signals.

Secondly, although p53 acts as an aggregator of incoming stress information, the classical mono-targeted approach ignores the context of p53-independent signaling pathways. Studies by Galit Lahav’s group have demonstrated that the selection of cellular programs differs fundamentally upon exposure to gamma irradiation versus ultraviolet stress, as the latter simultaneously activates concurrent p53-independent signaling cascades [69]. The integration of these independent signals allows the cell to maintain trajectory flexibility when transitioning between stable attractors, whereas isolated pressure on a single node deprives the system of this adaptability.

Thirdly, a fundamental limitation of mono-targeted stabilization is the high rate of *TP53* somatic mutagenesis in tumor populations. The design of Nutlins is tailored to block the degradation of the wild-type protein; however, in over half of all malignancies, p53 harbors missense mutations localized within its central domain. The application of MDM2 inhibitors in such contexts ensures the expected supraphysiological accumulation of the macromolecules in the nucleus. Yet, due to profound spatial conformational disruptions or the loss of key contact amino acid residues, the mutant protein is physically incapable of effective cooperative interaction with specific DNA binding sites. Consequently, brute-force pharmacological intervention merely leads to the accumulation of functionally inert p53, which fails to initiate target gene transcription but remains capable of exerting a dominant-negative effect on any remaining native subunits.

The forced maintenance of supraphysiological p53 levels leads to the collapse of the dynamic range across the entire informational system. In the absence of tissue-specific cofactors and with the complete suppression of temporal (pulsatile) modulation, p53 loses its operational selectivity. A persistent analog plateau of protein concentration is a necessary, but not sufficient, condition for initiating apoptosis [65,69]. The forced accumulation of the regulator without natural upstream or downstream signals leaves the p53 informational signal biophysically incomplete—for instance, lacking the critical phosphorylation at Ser46 required for selective affinity to the *BAX* and *PUMA* promoters [72]. At the transcriptional level, this manifests as a loss of selectivity and the simultaneous triggering of alternative, functionally conflicting programs. Thus, informational chaos arises not so much from the absolute amplitude of the incoming stimulus, but rather from the complete destruction of the signal’s temporal structure, which initiates stochastic expression cascades that bypass evolutionary regulatory filters.

This systemic distortion accounts for the numerous anomalous and unpredictable effects observed during the experimental application of Nutlins. The plateau-like p53 signal demarcates a bifurcation point, the exit from which leads to two alternative terminal attractors: apoptosis or cellular senescence [71]. Contrary to expectations, direct p53 stabilization by Nutlins frequently results not in cell death, but in the induction of the senescence program [98,99,100,101,102]. In the context of the elevated buffering capacity of anti-apoptotic proteins (such as Bcl-2, Bcl-xL, and Mcl-1) characteristic of solid tumors, an isolated p53 plateau cannot overcome the threshold resistance of the mitochondrial membrane and is inevitably captured by the senescence attractor [65]. For example, Shen et al. demonstrated that p53 induction via Nutlin-3a does not merely drive A549 and MCF7 cell lines into senescence, but irreversibly locks this program in tetraploid subpopulations even after the complete removal of the therapeutic agent from the medium [103].

Instead of the apoptotic eradication of the malignant cell mass, the transition into the senescence attractor triggers the senescence-associated secretory phenotype. Through the secretion of this cytokine and chemokine cocktail, these cells induce chronic inflammation in the microenvironment and stimulate the subsequent metastasis of adjacent surviving populations [104,105]. Clinical trials and in vitro studies corroborate this sharp divergence in responses depending on the histological tissue type. While hematological malignancies (lymphomas, leukemias)—which possess an inherently low defensive threshold—exhibit high sensitivity to apoptotic stimuli upon Nutlin administration, solid tumors more frequently respond merely with temporary and reversible cell cycle arrest or a shift into senescence [63]. Conversely, the most pronounced manifestation of these inhibitors’ off-target activity in the clinic has been severe systemic toxicity against healthy tissues with a low resistance threshold, typically presenting as profound thrombocytopenia [106].

Ultimately, the contradictory effects observed in clinical settings serve as direct confirmation that the brute-force pharmacological stabilization of a central regulatory hub—without regard for its dynamic architecture and tissue-specific context can lead to a total collapse of the control system. The attempt to exert linear pressure on a non-linear, multi-layered network without modulating its qualitative PTM code and without synergistically lowering the downstream mitochondrial barriers results in the destruction of the signal’s informational structure. This transforms a targeted therapeutic intervention into a stochastic process with a high probability of generating unforeseen pathological outcomes.

### 9.2. Targeted Protein Degradation (Direction 2)

The transition from the classical “lock-and-key” occupancy of active sites to the forced disposal of target proteins has radically altered the control dynamics of the p53 network [107,108]. This approach employs bifunctional degrader molecules (PROTACs), which bridge the MDM2 protein with intracellular E3 ubiquitin ligases (such as cereblon or the von Hippel-Lindau protein), thereby initiating its proteasomal degradation [109,110]. An alternative strategy utilizes hydrophobic tagging: the attachment of a bulky lipophilic moiety to the inhibitor mimics target denaturation, coercing the cell’s endogenous quality control systems into autonomously destroying the tagged MDM2 [111]. Both classical Nutlins and higher-affinity next-generation inhibitors, including idasanutlin and spiropyrrolidine derivatives, are successfully utilized as targeting ligands within these molecules [108,109].

The primary systemic advantage of targeted degradation is the shift from stoichiometric blockade to an iterative catalytic cycle. A single degrader molecule can sequentially destroy multiple molecules of the regulatory protein, elegantly circumventing the fundamental vulnerability of classical therapy: the activation of the negative feedback loop and the excessive compensatory synthesis of de novo MDM2, which necessitates continuous dose escalation under standard inhibition [107,109]. The potent, transient pulses of p53 liberation generated by degraders induce a rapid surge in its concentration, a profile biophysically much closer to the cell’s natural oscillatory dynamics under stress. Consequently, this class of drugs demonstrates a substantial amplification of the cytotoxic effect—achieving up to a 150-fold superiority over their parent inhibitors in certain leukemia models [110]. Furthermore, the selective recruitment of E3 ligases that exhibit low basal activity in healthy blood cells affords the protection of the bone marrow from severe thrombocytopenia [109].

Notably, the profound modulation of the p53 network via MDM2 degraders has opened unexpected therapeutic windows beyond oncology [110,112]. Controlled pulses of MDM2 degradation activate the expression of mineralization markers and trigger the bone tissue maturation cascade, which is being investigated as a promising approach in regenerative medicine for the treatment of severe injuries and osteoporosis [113].

Nevertheless, the methodology confronts fundamental limitations inherent to the p53 network topology itself. The artificial accumulation of the protein does not guarantee its correct qualitative encoding at the level of post-translational modifications. In the absence of genuine genotoxic damage and the activation of afferent kinase cascades, the p53 signal remains “incomplete,” frequently leading not to apoptosis, but to the system’s transition into the irreversible senescence attractor. Moreover, the targeted destruction of MDM2 loses all therapeutic rationale in the presence of missense mutations within p53 itself, where its DNA-binding domain is physically compromised. Under such conditions, the capacity of certain degraders to liberate the evolutionary p53 homolog—the TAp73 protein—assumes critical importance. Released from MDM2-mediated repression, this related transcription factor seizes transcriptional control and successfully initiates the malignant cell death program, bypassing the damaged primary hub and inaugurating an entirely novel therapeutic strategy [114].

### 9.3. Pharmacological Chaperones (Direction 3)

While targeted degradation strategies are effective in the context of wild-type p53, the concept of pharmacological chaperones is specifically aimed at the conformational repair of the mutant protein. Over half of all malignant neoplasms harbor missense mutations within the DNA-binding domain of p53 [88,89,90]. A hallmark example is the Y220C mutation: the substitution of a bulky tyrosine residue with a smaller cysteine creates a superficial hydrophobic crevice. This structural defect renders the protein thermodynamically unstable at physiological temperatures, thereby abolishing its DNA-binding capacity. Conformational chaperones are small molecules designed to selectively occupy this pocket; they act as a structural scaffold, restoring the thermodynamic stability and native functionality of p53 as a fully competent transcription factor [115,116].

A prominent outcome of such in silico structure-based drug design is rezatapopt (PC14586), a first-in-class ligand targeting the mutant Y220C pocket. Biophysical assays demonstrate that the tight binding of this chaperone increases the melting temperature (*T_m_*) of the damaged p53 domain from an unstable 34 °C to the physiological norm of 42 °C [115]. The systemic advantage of pharmacological chaperones lies in the fact that they do not require de novo protein synthesis. Instead, they directly “reformat” the massive, pre-existing intracellular pool of unfolded mutant p53, locking it into a stable, functional conformation. The geometrical restoration of the DNA-binding interface reinitiates the transcription of canonical target genes, including the cell cycle arrest regulator *CDKN1A* (*p21*) and the feedback control marker *MDM2* [116]. This conformational repair strategy has demonstrated preliminary clinical activity in early-phase trials for both solid tumors and acute myeloid leukemia [117].

An alternative approach to conformational restoration is eprenetapopt (APR-246). Unlike rezatapopt, which targets a highly specific binding pocket, eprenetapopt is a prodrug that converts intracellularly into a reactive component. This active metabolite covalently binds to exposed cysteine residues within the core of mutant p53, thereby inducing its refolding into a wild-type-like conformation [118,119,120].

A significant merit of pharmacological chaperones is their high structural selectivity for the mutant conformational state. Healthy cells harboring native, wild-type p53 physically lack these defective conformational pockets, meaning there are no direct structural targets for these chaperones in normal tissues. While this mechanism minimizes the risk of target-mediated toxicity in healthy organs—directly addressing the primary systemic issue inherent to the non-selective stabilization of p53 by Nutlins—it is important to note that mutation selectivity does not imply complete tissue selectivity. This targeted approach does not entirely eliminate the potential for pharmacokinetic, metabolite-mediated, or target-independent off-target toxicities typical of small-molecule therapeutics.

Nevertheless, a systemic analysis reveals substantial limitations to this biophysical approach:

- Narrow spectrum of activity: Rezatapopt exclusively rectifies the Y220C defect, which accounts for merely 1.8% of all cancer cases. It remains entirely blind to hundreds of other known *TP53* missense mutations and is fundamentally useless in the context of *TP53* gene deletions.
- Clonal evolution: Tumor heterogeneity drives the rapid emergence of resistance. Single-cell sequencing has demonstrated that the targeted elimination of the Y220C-mutant clone instantaneously relieves intratumoral competition [117]. In response to this selective pressure, minor clones harboring alternative mutations (e.g., *TP53*-P151A or *NRAS*) rapidly expand to dominate the population.
- Signal incompletion: The biophysical reactivation of p53 does not guarantee the induction of apoptosis. While conformational repair restores the genomic reading interface, it does not compensate for the deficit in afferent chemical encoding. In tumor cells, the upstream pathways responsible for the correct phosphorylation of the hub are frequently compromised. In the absence of specific post-translational modifications (PTMs), the transcriptional signal remains incomplete: the restored p53 may stall at the cell cycle arrest stage or drive the system into the dead-end attractor of senescence. For this reason, in a clinical setting, chaperones realize their optimal therapeutic potential only when combined with other agents.

### 9.4. Activation of Evolutionary Homologs as Alternative Molecular Switches (Direction 4)

In cases where the *TP53* gene is completely deleted or its protein product is irreversibly damaged (e.g., following the emergence of secondary resistance to chaperones), direct pharmacological targeting of the central hub becomes impossible. Under such conditions, a viable systemic solution involves rerouting information flows through evolutionary backup nodes: the p63 and p73 proteins. As noted earlier (in the context of the Liskov Substitution Principle), these homologs share a highly conserved DNA-binding domain with p53, enabling them to recognize and activate analogous genomic regulatory elements [121]. Furthermore, they are exceedingly rarely subjected to somatic mutations during oncogenesis [122].

The transactivation-competent isoforms of these proteins (specifically, TAp63 and TAp73) are negatively regulated by MDM2 and its structural analog, MDMX [121]. These negative regulators bind to the transactivation domains of the homologs, suppressing their transcriptional activity without necessarily triggering their proteasomal degradation [121,123]. In p53-deficient cells, the MDM2/MDMX-p73 axis plays a critical, p53-independent role in cell cycle regulation and the maintenance of E2F-family transcription factor activity (primarily E2F1). This functional independence establishes p73 as a crucial and autonomous therapeutic target [123]. Consequently, the pharmacological inhibition of the MDM2-MDMX heterodimer can successfully liberate these intact backup controllers.

This approach is particularly significant for tumors harboring mutant p53 with Gain-of-Function (GoF) properties [122]. Such mutant proteins can form inhibitory complexes—often through co-aggregation—with p73 and p63, thereby completely abolishing their tumor-suppressive activity [122,124]. A targeted engineering solution to this structural problem involves the use of specific small molecules, such as RETRA [124]. This compound selectively disrupts the inhibitory complex formed between mutant p53 and p73 [122,124]. The subsequent release of free TAp73 leads to the transcriptional reactivation of classical target genes (such as *CDKN1A/p21* and *BBC3/PUMA*) and the induction of apoptosis. In initial preclinical models—specifically in vitro mutant p53-bearing human cell lines and mouse xenografts—RETRA demonstrated high selectivity, promoting tumor suppression while exhibiting minimal toxicity to normal fibroblasts [124]. However, it is essential to emphasize that these observations are derived strictly from preclinical settings, and such targeted selectivity cannot yet be definitively extrapolated to general human therapeutic safety. Thus, the pharmacological alleviation of this repression enables the evolutionary homologs to assume transcriptional control, functionally compensating for the critical failure within the cell’s signaling network architecture. However, the exploitation of these homologs encounters several systemic barriers:

- Molecular buffering: Endogenous dominant-negative isoforms (ΔNp63/ΔNp73) can act as a stoichiometric buffer, binding and sequestering the therapeutically liberated active variants [121,122].
- Insoluble co-aggregation: GoF p53 mutants frequently form dense, amyloid-like aggregates that tightly trap the homologs. Extracting them using small molecules alone—without concurrent aggregation inhibitors—is exceedingly difficult [122].
- Signal incompletion: The physical release of the homolog does not inherently guarantee signal initiation. For instance, TAp63α exists as an autoinhibited dimer that requires a specific, sequential phosphorylation cascade (e.g., via Chk2 and CK1) to assemble into a fully functional tetramer [121].
- Viral degradation: Finally, in virus-associated malignancies, viral oncoproteins (such as HPV E6) can directly target p63 and p73 for rapid proteasomal degradation, completely abrogating the therapeutic potential of this bypass route [122].

### 9.5. Synergistic Activation and Topological Barrier Lowering (Direction 5)

As detailed during the analysis of Layer 5, the biological response to p53 activation is strictly governed by a threshold mechanism [65]. The isolated hyperactivation of the hub (for instance, via the MDM2 antagonist idasanutlin) is frequently insufficient to overcome the buffering capacity of anti-apoptotic proteins. Consequently, the cell does not succumb to apoptosis, but merely transitions into the safe attractor of temporary cell cycle arrest (the G1 phase) [65].

To forcibly switch the system from temporary arrest into irreversible apoptosis, a mechanism of profound pharmacological synergy—specifically, apoptotic priming—is often required. This combination is more accurately described as a cooperative pathway dependence. This synergy is achieved through the combined application of p53 activators and BH3 mimetics (such as the Bcl-2 inhibitor venetoclax) [125]. Venetoclax topologically lowers the apoptotic resistance barrier, instantaneously neutralizing the kinetic delay associated with the transcriptional response to MDM2 inhibition and drastically accelerating cell death [125]. As demonstrated in models of acute myeloid leukemia, p53 activation suppresses the MAPK signaling cascade, thereby unleashing the activity of GSK3 kinase. This kinase subsequently targets the key anti-apoptotic protein Mcl-1 for rapid proteasomal degradation [126]. This accelerated depletion of Mcl-1 strips the tumor of its primary defense mechanism against venetoclax [126].

However, a systemic limitation of this synergism is its fundamental futility in the presence of profound mutations within the p53 DNA-binding domain; without a functionally intact central signal generator, the downstream cascade leading to Mcl-1 degradation cannot be initiated [126]. Furthermore, dual pharmacological pressure establishes a novel point of failure within the network: the tumor can undergo clonal evolution, acquiring secondary resistance mutations directly within the downstream mitochondrial effectors themselves (for example, within the Bcl-2 protein).

### 9.6. Targeting Redundant Nodes (Direction 6)

In conditions where the *TP53* gene is completely deleted or mutated, attempts to reactivate it directly lose their therapeutic rationale. However, the loss of p53 signifies the abrogation of control over the G1/S checkpoint, which compels the tumor to shift a significant burden of genomic integrity surveillance onto a redundant backup node—the G2/M checkpoint, primarily operated by the WEE1 kinase [127].

This creates a state of genetic synthetic lethality, classically defined as a condition where the simultaneous loss of two distinct genes or pathways (in this case, p53 and WEE1) results in cell death, whereas the loss of either alone remains viable. However, it is critical to emphasize that the p53/WEE1 synthetic lethal relationship is highly context-dependent rather than universally dictated solely by TP53 status. The therapeutic vulnerability to WEE1 inhibition relies heavily on the baseline level of oncogene-induced replication stress and the specific epigenetic background of the tumor [127]. When these contextual conditions are met, targeted inhibition of WEE1 (for example, using MK-1775/adavosertib) deprives the tumor of this final failsafe [128]. P53-deficient cells harboring DNA damage lose their capacity to arrest for repair and are forcibly driven into mitosis, culminating in mitotic catastrophe [127].

This mechanism is executed with particular elegance during combinatorial intervention: partial inhibition of WEE1 destabilizes the Hipk2 kinase (a negative regulator of the Wip1 phosphatase), which potentiates Wip1-dependent cell death exclusively in p53-negative tumors [128]. The application of even subtoxic doses of MK-1775 in combination with Wip1 induction allows for a 40% reduction in the effective dose of chemotherapy (cisplatin) [128]. Moreover, such an architectural rewiring of signaling pathways protects normal cells (harboring wild-type p53), thereby minimizing the overall cytotoxicity of the treatment in healthy tissues [128].

Ultimately, this approach epitomizes a fundamental paradigm shift: transitioning from attempts to repair a broken central hub toward the targeted destruction of the redundant architecture upon which the tumor has been forced to rely.

### 9.7. Pharmacological Sheltering of the System (Direction 7)

If the sustained hyperactivation of the central hub frequently leads to systemic chaos, a paradoxical yet structurally sound engineering approach involves temporarily insulating the healthy system from therapeutic stress. The primary dose-limiting factor in classical genotoxic radio- and chemotherapy is the massive, p53-dependent apoptosis of rapidly dividing healthy cells. Conversely, tumor cells that have already lost functional p53 succumb to such therapies via p53-independent mechanisms (e.g., through mitotic catastrophe).

To achieve this “pharmacological shelter,” two diametrically opposed network manipulations can be employed: deactivating the hub or forcing it into a safe diagnostic mode.

The first strategy relies on the temporary complete deactivation of the hub. The application of small synthetic molecules, such as pifithrin-alpha (PFT-α), reversibly blocks the transcriptional activity of p53 and abrogates p53-dependent apoptosis [129]. This allows healthy tissues to endure the peak of exogenous cytotoxic stress. Following the clearance of the inhibitor, normal homeostatic control is restored, and this transient suppression of the hub does not elevate the incidence of de novo tumor formation [129].

The second, inverse strategy leverages the hub to **force a systemic pause** via non-genotoxic p53 activation (an approach known as cyclotherapy). Although MDM2 antagonists like Nutlins are primarily designed to eradicate tumors, they possess a profound cytoprotective potential for normal tissues [130]. By stabilizing wild-type p53, Nutlin selectively drives healthy cells—such as bone marrow hematopoietic stem cells—into a reversible cell cycle arrest [131]. This effectively locks the healthy network into a safe, dormant attractor, shielding it from the lethal damage of phase-specific chemotherapies (such as taxol) that target actively dividing cells [132].

Crucially, this growth arrest is strictly reversible upon drug withdrawal, allowing normal cells to safely resume proliferation and significantly mitigating treatment-related complications like myelosuppression [95]. Furthermore, Stine et al. demonstrated that this network modulation confers bone-sparing effects during cisplatin therapy, preserving healthy tissue integrity without compromising the drug’s tumor-suppressive efficacy [133].

While the immediate clinical translation of Nutlins specifically for cytoprotection may be constrained by the off-target systemic toxicities discussed in earlier sections, the mechanistic engineering principle remains highly relevant. Whether by temporarily shutting down the central controller (PFT-α) or intentionally locking it into a reversible “Stay” attractor (Nutlin), intentionally overriding the hub’s default dynamic responses provides a critical window to protect the healthy macro-system from extreme exogenous noise.

### 9.8. In Silico and Allosteric Network Design (Direction 8)

Because p53 operates as a super-hub, direct pharmacological targeting of its primary DNA-binding interface is practically impossible. Consequently, the vector of modern drug discovery is shifting toward allosteric modulation—the design of molecules that bind to the protein at one distinct site but dynamically alter its function at a distance [134].

For a long time, computational biology committed a reductionist error by focusing exclusively on the rigid, highly structured DNA-binding domain (DBD) of p53. However, p53 belongs to the class of Intrinsically Disordered Proteins (IDPs). Its N- and C-terminal regulatory domains lack a fixed 3D structure, yet they construct the multi-layered allosteric scaffold of the protein [135]. From the perspective of our proposed system architecture, this vividly illustrates the interaction between Layer 1 and Layers 2 and 3: chemical signals and modifications of flexible IDP regions (Layer 1) are allosterically transmitted to isolated functional ports (Layer 2), ultimately integrating into the monolithic decision-making network of the entire monomer (Layer 3). Molecular dynamics simulations of full-length p53 have proven that these flexible “tails” dynamically dictate both the destructive impact of mutations and the rescue mechanisms of therapeutic drugs [135]. For instance, the direct interaction of the disordered C-terminal domain (CTD) with DNA catalyzes a structural reorganization of the protein core: the S6-S7 loop shifts into a closed conformation, while the L1 loop approaches the H2 helix, physically stabilizing the DNA-protein complex [49].

The reactivation of mutant p53 (e.g., the Y220C mutation) by small molecules such as PK11000 does not occur via a massive, mechanical refolding of the protein [135]. Instead, the drug acts with far greater subtlety: it restores a disrupted network of electrostatic interactions and hydrogen bonds that propagates throughout the entire full-length protein, encompassing its disordered IDP regions [135].

Such allosteric modulation unveils an unprecedented opportunity: not merely to reactivate a damaged hub, but to purposefully reprogram its transcriptional selectivity. Subtly altering the spatial geometry of the DNA-binding interface from a distance theoretically allows for the artificial manipulation of affinity constants (*K_D_*, see Table 4). This dynamic tuning could enhance the affinity of p53 for historically low-affinity pro-apoptotic promoters (such as *BAX* or *PUMA*) while simultaneously attenuating its binding to cell cycle arrest genes (e.g., *CDKN1A*). In the context of our systemic model, this equates to the pharmacological capacity to “rewrite” the Layer 5A routing code, forcibly directing the tumor cell’s trajectory straight into the terminal apoptosis attractor, completely bypassing the kinetic delay of protective cell cycle arrest.

To track these concealed allosteric signals, modern computational algorithms are employed, which can be conceptualized through intuitive biological analogies:

- Differential Contact Network Analysis (dCNA): This operates akin to mapping “social networks” within the molecule. The algorithm tracks how communities of interacting amino acid residues reorganize upon drug binding or mutation, successfully revealing hidden signal transduction pathways [136].
- Protein Sector and Heat Core Analysis (HCA): This approach visualizes how a wave of energetic perturbation “diffuses” from the molecule’s binding site to the distant DNA interface, analogous to the propagation of thermal energy through a physical object [134].

Specifically, the application of dCNA has already demonstrated robust in silico predictive power in identifying modular rearrangements and hidden allosteric communication pathways during ligand binding [136]. Nevertheless, a crucial caveat must be established: to date, the described approaches leveraging generative neural networks, molecular dynamics, and topological analysis remain predominantly computational models. Despite their immense theoretical value, de novo small molecules designed specifically via these algorithms for the allosteric control of p53 have yet to undergo large-scale, robust experimental validation.

## 10. Evolutionary and Genetic Models of Successful p53 Network Tuning

Attempts at direct pharmacological intervention on the p53 network frequently result in systemic failures: the excessive stimulation of this central hub induces informational chaos and premature tissue depletion, whereas its systemic suppression strips the cell of its primary tumor-suppressive defense. However, both the natural world and targeted genetic experiments provide fascinating biological inspiration for how the architectural vulnerabilities of a “God Object” and a Single Point of Failure (SPOF) might conceptually be resolved without disrupting physiological homeostasis.

### 10.1. “Super-Mice” and Elephant Retrogenes

Classical transgenic models harboring constitutively active or mutant p53 exhibit high cancer resistance, but pay a severe physiological toll characterized by a catastrophic acceleration of aging and premature organ atrophy. The root cause of this toxicity lies in the disruption of basal regulation: a continuous, unmodulated signal forcibly drives the cells into the terminal attractors of senescence.

A fundamentally different systemic response is demonstrated by “Super p53” mice, which carry additional copies of the *Trp53* gene within large genomic fragments that preserve their native regulatory elements [137]. In these models, the basal level of p53 remains low due to the normal functioning of the MDM2-mediated negative feedback loop; however, upon the onset of genotoxic stress, the system generates a markedly stronger and faster pulsatile response [137]. This ensures the enhanced elimination of damaged cells and significant resistance to chemical carcinogenesis, all while preserving a natural lifespan and normal aging kinetics [137].

An example of the natural scaling of this strategy is observed in elephants (*Loxodonta africana* and *Elephas maximus*), whose massive body size and extended lifespan theoretically incur a heightened risk of malignant transformation (Peto’s paradox) [138]. Proboscidean evolution resolved this challenge through the massive expansion of the *TP53* genetic repertoire: their genome contains a single canonical copy and 19 retrogenes (*TP53RTG*) that arose via segmental duplications in parallel with the evolutionary increase in body mass [138].

A subset of these retrogenes is actively transcribed and translated into truncated protein products (for instance, TP53RTG12) [138]. Lacking the classical DNA-binding and tetramerization interfaces, these proteins do not function as direct transcription factors, yet they retain the capacity to heterodimerize with canonical p53 [138]. This interaction effectively shields p53 from ubiquitination by the MDM2/MDMX complex, establishing a mobile pool of pre-assembled protein and ensuring a hypersensitive trigger for apoptosis even at minimal doses of genotoxic stress [138].

### 10.2. Peripheral Offloading in the Naked Mole-Rat

An alternative evolutionary adaptation is observed in the naked mole-rat (*Heterocephalus glaber*)—a small rodent boasting a record lifespan (exceeding 30 years) and exceptional resistance to spontaneous oncogenesis [139,140,141]. Rather than constantly overloading the central p53 transcriptional processor, the naked mole-rat redistributes its defensive burden to the network periphery via a mechanism known as early contact inhibition (ECI) [139].

Naked mole-rat fibroblasts arrest proliferation at a relatively low monolayer density, establishing a dual-tiered control system [139]:

- First tier (early response): Governed by the overexpression of the p16 protein, which triggers cell cycle arrest upon initial intercellular contact.
- Second tier (reserve barrier): Maintained by the canonical contact inhibition inducer, p27, which deploys only if the primary defensive line is breached.

The extracellular trigger for this early inhibition is the secretion of an anomalously high-molecular-mass hyaluronic acid (HMW-HA, 6–12 MDa). This polymer is synthesized due to specific mutations within the active site of the HAS2 enzyme and degrades slowly owing to reduced hyaluronidase activity [140]. Signal transduction from HMW-HA via the CD44 receptor and the NF2 tumor suppressor strictly confines the cells within a safe operational basin; conversely, the enzymatic degradation of this extracellular shield by hyaluronidases renders the cells susceptible to oncogenic transformation [140].

Concurrently, the central p53 hub in naked mole-rat cells exhibits kinetics highly atypical for mammals: its half-life exceeds that of its murine and human orthologs by more than an order of magnitude. Furthermore, a substantial fraction of the protein is constitutively localized within the cell nucleus even in the absence of exogenous stress [141]. Despite this constant nuclear “surveillance,” this pre-assembled p53 pool does not trigger basal apoptosis; however, upon detecting DNA damage, it instantaneously induces the transcription of canonical target genes (such as *CDKN1A*/*p21*), rigorously maintaining genomic stability [141].

Together, these natural adaptations and genetic models highlight two fundamental design principles that can serve as biological inspiration for the regulation of biological super-hubs:

- Preservation of dynamic feedback control: Increasing the hub’s gene dosage is therapeutically viable only if its native regulatory circuitry is meticulously preserved (as seen in “Super-mice” and elephants). This prevents the systemic trajectory from slipping into the toxic terminal attractor of premature senescence [137,138].
- Peripheral offloading of the central node: The deployment of multi-layered external barriers (e.g., ECI, HMW-HA, and redundancy via p16/p27) effectively filters out oncogenic “noise” long before the informational load can overwhelm the system’s central Single Point of Failure—the p53 protein [139,140].

Ultimately, these natural blueprints suggest that the inherent vulnerability of a biological “God Object” is best mitigated not through its forced pharmacological hyperactivation, but through architectural decentralization and the precise, dynamic tuning of the entire signaling network. It must be emphasized, however, that evolutionary adaptations represent whole-organism, long-term biological solutions developed over millions of years. Therefore, they cannot be treated as direct experimental validation that an analogous short-term pharmacological intervention will be safe or immediately effective in human cancers. Instead, they provide profound conceptual support and biological inspiration for our hypothesis. Translating these evolutionary strategies into clinical practice represents a fundamental challenge for systemic oncology.

## 11. Mapping Pharmacology onto the p53 Network Architecture

The therapeutic strategies reviewed herein demonstrate that the successful control of the p53 network is highly challenging without accounting for its hierarchical, multi-layered organization. As detailed in Table 6, contemporary pharmacological approaches are classified according to the specific layer of the systemic architecture (Figure 3) they target. This stratification elucidates the underlying topological patterns governing their clinical successes and highlights the systemic vulnerabilities responsible for their potential failures.

Analysis of this stratification reveals a fundamental pattern in systems pharmacology: interventions restricted to the lowest architectural levels (e.g., simple stoichiometric blockade at Layer 2) frequently encounter robust systemic resistance, as they fail to account for higher-order network dynamics and feedback loops. In contrast, the most promising therapeutic avenues involve either the synergistic manipulation of multiple network layers (e.g., combining Layer 2 liberation with Layer 5 barrier reduction) or the deliberate exploitation of the tumor’s altered topology (targeting redundant pathways at Layer 6). Ultimately, this paradigm shift—from the linear inhibition of single protein targets to the dynamic, multi-layered modulation of the entire signaling architecture—represents the core thesis for the future of p53-targeted oncology.

## 12. The Translational Barrier: Why Systemic Strategies Still Fail in the Clinic

The network and allosteric strategies described above demonstrate outstanding results in silico and in preclinical models; however, their integration into routine clinical practice confronts a formidable barrier. The transition of these molecules into a living organism exposes vulnerabilities inherent to both the chemical agents themselves and their selected biological targets. For instance, while the compound PK11000 successfully restores the conformation of the Y220C mutant in vitro, it remains unsuitable for clinical application due to its non-specific covalent binding with the thiol groups of off-target proteins, which induces unacceptable systemic toxicity [134,135].

Concurrently, monotherapy directed at a single point of vulnerability (e.g., the targeted inhibition of WEE1 intended to exploit synthetic lethality following p53 loss) frequently fails to yield the requisite therapeutic efficacy. The highly plastic tumor network rapidly circumvents this localized blockade by activating parallel signaling pathways (such as the ATR/CHK1 cascade). Furthermore, the drug itself inflicts severe damage on healthy, rapidly dividing tissues, thereby critically narrowing the therapeutic window [127]. Attempts to compensate for these limitations through the empirical selection of drug combinations often lead not to true therapeutic synergy, but to the mere additive accumulation of toxicities. This failure occurs fundamentally because such empirical regimens are not grounded in rigorous mathematical forecasting of the entire biochemical network’s dynamic response [127].

To clearly delineate the current translational landscape and separate preclinical concepts from clinical realities, Table 7 summarizes the key pharmacological strategies discussed in this review. This stratification highlights the specific dependencies of each modality on the TP53 mutational status, their current stages of clinical development, and the primary systemic bottlenecks—whether target-mediated toxicities or rapid clonal evolution—that currently limit their universal application.

## 13. Hypothesis and Strategic Solutions: A Perspective Framework

The evidence synthesized in this review indicates the necessity for a conceptual paradigm shift in our approach to cellular super-hubs. Historically, the classical “lock-and-key” pharmacological model achieved monumental clinical success in infectious diseases because it exploited an inter-systemic conflict—targeting unique pathogenic proteins completely absent in the human host. In contrast, oncology represents an intra-systemic failure where malignant cells utilize the exact same molecular hardware as healthy tissues. Consequently, applying simple linear interventions (such as direct MDM2 blockade) to a shared central regulator inevitably encounters severe systemic toxicity and adaptive network plasticity.

To resolve this intra-organismal challenge, classical network medicine (which traditionally models interactomes as “flat” horizontal graphs and suggests seeking bypass routes via adjacent nodes) must be expanded. By deconstructing p53 into a seven-layer hierarchy, we introduce a new, vertical dimension (the “depth” of the node itself) into network pharmacology, where Layers 1–4 describe the internal signal compilation within the protein molecule, and Layers 5–7 define its external realization in the tissue.

The emergence of this volumetric coordinate system allows us to formulate our primary hypothesis and pose a fundamental question to modern bioinformatics and medicinal chemistry: which strategic vector of super-hub management is optimal—intervention “by depth” (meticulous multi-layered tuning of a single central p53 hub accounting for all seven strata of its architecture) or intervention “by breadth/horizontally” (abandoning the central hub to strike diverse bypass nodes within a single chosen network level, as classical network theory suggests)?

We posit that the answer is determined by a fundamental “complexity threshold” (a controllability horizon) of the signal, which directly depends on tissue nature and the target’s network integration degree:

- For simple peripheral proteins with low connectivity (*C_a_*), neither deep-level penetration nor horizontal bypass routing is required; classical point-targeted “lock-and-key” interventions remain entirely sufficient.
- The “depth” vector (focusing on a single hub across seven layers): A paramount task for future research is defining the exact tissue boundaries—is it possible at all (and in which specific tissue types) to achieve a deterministic cure by targeting the p53 hub alone, but doing so comprehensively across its internal vertical axis (e.g., combining domain restoration at Layer 2 with allosteric promoter selectivity tuning at Layer 3/5A in tumors with low protective thresholds, such as leukemias)?
- The “horizontal” vector and orthogonal synthesis (focusing on diverse nodes): In tissues with high buffering capacity (e.g., solid carcinomas) or facing irreversible hub damage, signal complexity may exceed the mathematical limit of single-point controllability. In such cases, attempting to modulate only p53 (even considering its internal architecture) will merely generate informational chaos or induce senescence. This necessitates either a strictly horizontal bypass at a specific layer (attacking independent nodes like WEE1 or p73 homologs) or an orthogonal, multi-point strike across different strata simultaneously (e.g., hub reactivation at Layer 2 combined with mitochondrial Bcl-2 barrier suppression at Layer 5B).

Thus, because fundamental tissue topology differs radically, no universal formula exists—the future lies in identifying tissue- and tumor-specific “local optima” that balance vertical hub modulation with horizontal network interception. As insightfully noted during peer review, we present the search for such optima not as a ready-to-use clinical algorithm, but as a perspective research trajectory. Translating this concept into quantitative in silico modeling will require defining strict tumor-architecture parameters:

- State Variables (Inputs): Mapping the fundamental wiring of a specific tissue across layers (e.g., the nature of the p53 defect at Layer 2, basal PTM profiles at Layer 1, Bcl-2/Mcl-1 intrinsic buffer capacities at Layer 5B, and checkpoint status at Layer 6). This will determine whether “vertical” control of a single hub remains viable or if the controllability limit has been crossed.
- Objective Function: Modeling perturbations aimed at mathematically minimizing the energetic barrier to shift the cell into the terminal apoptosis attractor (Layer 6) while avoiding the irreversible senescence trap.
- Systemic Constraints: Establishing rigid upper bounds for pharmacological pressure to preserve the homeostasis of healthy, low-resistance tissues (such as bone marrow), thereby avoiding dose-limiting collateral toxicity.

Once these fundamental tissue-specific boundaries (dictating when “in-depth” single-hub targeting suffices versus when horizontal multi-point bypass is mandatory) are mathematically delineated, individual patient-specific multi-omics profiles will serve as the ultimate clinical superstructure to achieve maximum therapeutic selectivity. Ultimately, the seven-layer deconstruction of p53 presented here may establish a conceptual matrix: future studies must determine how vertical depth and horizontal connectivity interrelate for other critical cellular super-hubs (such as MYC, RAS, or NF-κB).

## 14. Literature Selection and Conceptual Methodology

### 14.1. Conceptual Framework and Purposive Literature Sampling

Given the exceptionally broad and interdisciplinary scope of this article—spanning evolutionary biology, structural crystallography, complex network theory, and clinical pharmacology—this manuscript was designed as a conceptual, theory-building narrative review rather than a quantitative systematic review. Conducting an exhaustive, PRISMA-style systematic search of all p53-related literature is technically unfeasible due to the sheer volume of publications and falls outside the scope of constructing a structural framework.

To substantiate the proposed seven-layer volumetric architecture, a purposive literature sampling strategy was employed using databases such as PubMed/MEDLINE, Scopus, and Google Scholar. Sources were strictly curated based on their conceptual relevance to specific hierarchical levels. For the evolutionary and topological foundations, seminal works and highly cited historical analyses were prioritized. For biochemical modifications, domain interactions, and pharmacological strategies, we did not aim to compile an exhaustive catalog; rather, we selected representative landmark studies, key structural biology papers, and definitive clinical trial reports. These carefully chosen examples serve to robustly illustrate the specific mechanisms, inter-layer communications, and systemic vulnerabilities at each distinct architectural layer of the p53 network.

### 14.2. STRING Network Analysis Parameters

For the comparative interactome analysis presented in Section 3, data was retrieved from the STRING database (version 12.0) using the following exact parameters: the organism was restricted to Homo sapiens (query date: January 2026); the network type was set to the full STRING network; and active interaction sources were strictly limited to “Experiments” and curated “Databases” with a high confidence score (>0.700). The “Textmining” parameter was strictly disabled to reduce literature bias. To retrieve the total number of direct interacting partners, the maximum number of interactors in the first shell was set to “no more than 500”, without incorporating second-shell expansions. The topological data was subsequently exported for quantitative node counting.

## 15. Conclusions

In the present review, the architecture and function of the p53 tumor suppressor have been analyzed through the prism of systems engineering, employing a sequential deconstruction across seven hierarchical layers: from the basal post-translational code and local protein interfaces to global signaling networks and organismal homeostasis. This multi-layered examination highlights a core principle of systems pharmacology: the deeper a protein is integrated into the cellular informational network, the more challenging it becomes to regulate via single-target interventions. Specifically, the status of p53 as a densely connected cellular super-hub helps to explain why classical monotherapy within the reductionist “lock-and-key” paradigm frequently proves insufficient, primarily due to systemic toxicity and the adaptive plasticity of tumor networks.

To address this translational barrier, we have systematized alternative therapeutic strategies that target diverse strata of the cellular architecture. These include rerouting the signal through redundant homologs (p63/p73), topologically lowering apoptotic barriers (BH3 mimetics), exploiting context-dependent synthetic lethality by attacking compensatory nodes (WEE1), and the paradoxical temporary deactivation of the hub itself (pifithrin-α).

The conceptual viability of this signal load redistribution hypothesis is supported by evolutionary and genetic models. Specifically, the amplification of gene copy numbers in elephants and “Super p53” mice, alongside the delegation of the defensive barrier to multi-tiered contact inhibition mechanisms in the naked mole-rat, provide biological inspiration illustrating the potential efficacy of peripheral defense and the structural offloading of the central processor.

Finally, the proposed methodology of multi-level architectural analysis holds substantial potential as a perspective framework: because the difficulty of pharmacological control scales directly with a hub’s network centrality, the logic of systemic volumetric deconstruction demonstrated here for p53 can be conceptually extended to investigate other formidable, highly integrated cellular super-hubs (such as MYC, RAS, or NF-κB). While tissue-specific heterogeneity remains a major hurdle, the future advancement of oncopharmacology necessitates a paradigm shift from the pursuit of a single ideal target toward adaptive network governance—defining precise, multi-node combinatorial interventions capable of safely reprogramming the local homeostasis of a specific tumor without inflicting systemic damage upon healthy tissues.

## Figures and Tables

**Table 1:** Post-translational modifications (PTMs) of p53 as the foundational input code (Layer 1). This table outlines the primary chemical modifications that act as fundamental data inputs for the p53 network. By targeting specific amino acid residues, these dynamic PTMs collectively govern the stability, localization, and functional activation of the central controller in response to diverse cellular stresses.

Modification Type	Primary Target Residues	Functional Signal	References
Phosphorylation	Serine (Ser), Threonine (Thr)	Stress activation, stabilization	[24]
Acetylation	Lysine (Lys)	Transcriptional activation, DNA binding	[25]
Ubiquitination	Lysine (Lys)	Proteasomal degradation	[26]
Methylation	Lysine (Lys), Arginine (Arg)	Activation (K372) or Repression (K370/K382)	[27,28]
O-GlcNAcylation	Serine (Ser149)	Metabolic stabilization	[29]
Sumoylation	Lysine (K386)	Nuclear export and localization	[30,31]
Neddylation	Lysine (K320, K321)	Inhibition of transcriptional activity	[32,33]

**Table 2:** Structural and functional characterization of p53 autonomous domains. This table details the foundational molecular hardware (Layer 2) of the p53 network architecture. The autonomous modules are categorized by their primary interface type, distinguishing between domains that mediate external communication (e.g., binding to genomic response elements or co-activators) and those responsible for internal allosteric regulation, spatial organization, and oligomerization.

Domain Name	Interface Type	Primary Function	Key Structural Features	References
TAD (Transactivation)	External	Recruitment of co-activators (e.g., p300/CBP) and MDM2 binding.	Intrinsically disordered; “folding upon binding” mechanism.	[37]
DBD (DNA-Binding)	External	Sequence-specific recognition of genomic Response Elements (REs).	Complex β-sandwich scaffold with Zn-atom coordination.	[38,39]
OD (Oligomerization)	Internal	Provides the structural basis for tetramer formation.	α-helix and β-strand motif for dimer-dimer interface.	[40]
CTD (C-Terminal)	Internal	Allosteric regulation of DBD and non-specific DNA scanning.	Flexible, disordered region; major hub for Layer 1 modifications.	[25]
PRD (Proline-Rich)	Internal	Links TAD to DBD; essential for p53 stability and signaling efficiency.	PXXP motifs for SH3-domain protein interactions.	[41]

**Table 3:** Intramolecular network dynamics and inter-domain communication within the p53 architecture. This table summarizes key experimental and computational findings illustrating how distinct autonomous modules—including the Transactivation Domain (TAD), Proline-Rich Domain (PRD), DNA-Binding Domain (DBD), and C-Terminal Domain (CTD) cooperatively interact. These dynamic regulatory mechanisms, corresponding to the transition between Layer 2 (Protein Domains) and Layer 3 (Central Controller) of the system hierarchy, govern critical processes such as allosteric signaling, autoinhibition, and DNA target scanning.

Domains	Mechanism	Methods	Core Finding	Reference
CTD ↔ DBD	Facilitation of 1D linear diffusion (sliding) along DNA	Experimental	The CTD acts as an indispensable positive regulator of the core DBD; it enables the p53 molecule to linearly diffuse (slide) along the DNA backbone to efficiently locate target promoters, a search mechanism that is evolutionarily critical and cannot be substituted or stimulated by C-terminal phosphorylation or acetylation mimicking.	[45]
N-terminal Hinge (PRD extension) ↔ DBD	Cation-pi interaction and conformational stabilization.	Experimental	Trp91 in the N-terminal hinge region folds back onto the core domain to form a specific cation-pi interaction with Arg174. This structural feedback extends the functional boundaries of the DBD, increasing its melting temperature by up to 2 °C, modulating tetramer dissociation, and preventing non-native DBD-DBD interactions that trigger protein aggregation.	[46]
TAD (AD2 motif) ↔ PRD ↔ DBD	Phosphorylation-dependent electrostatic switch, autoinhibition, and conformational spacing	Experimental	The intrinsically disordered AD2 motif within the TAD undergoes dynamic intramolecular interactions with the structured DNA-binding surface of the DBD, competing directly with DNA. Constitutive phosphorylation of Thr55 (augmented by Ser46) increases the negative charge of the TAD, shifting the equilibrium toward a “closed,” autoinhibited state that completely abolishes positive DNA-binding cooperativity. The intervening Proline-Rich Domain (PRD) acts as a rigid “entropic bristle” that projects AD2 away from the DBD to prevent premature autoinhibition; its deletion facilitates the AD2-DBD interaction and forces the system into an autoinhibited state even without phosphorylation.	[47]
CTD (Conformational Ensemble)	Stochastic formation of Molecular Recognition Features (MoRFs).	Computational	The unbound p53-CTD is not a random coil but stochastically transitions through sets of minimal structural motifs (MoRFs) of 3–4 residues (e.g., asymmetric β-hairpins/bridges and short turns like _376_STS_378_ localized in its C-terminal half. These pre-formed elements provide structural complementarity to diverse external receptors (e.g., S100B, Sir2), regulating binding promiscuity via conformational selection followed by induced fit.	[48]
CTD ↔ DBD	Global allosteric network reconfiguration and direct IDR-DNA stabilization.	Computational	Microsecond-scale simulations and free energy landscapes reveal that while DNA binding locally alters the conformation of the DBD (specifically loops L1 and S6-S7), the intrinsically disordered CTD acts as a critical allosteric amplifier. The CTD directly interacts with the DNA molecule and actively reconfigures a distributed intra-protein allosteric network—spanning loop L1, loop S6-S7, β-strands S4 and S10, and helices H1 and H3—to thermodynamically lower free energy barriers and structurally promote high-affinity DBD-DNA binding.	[49]
CTD ↔ DBD	Sequence-context-dependent allosteric modulation and RE-driven conformational selection.	Experimental	The sequence-independent CTD dictates site-specific genomic binding in a context-dependent manner. The controller’s reliance on the CTD scales directly with the degree of deviation of the DNA response element (RE) from the canonical consensus sequence. For divergent, non-canonical genomic targets, the CTD stabilizes the protein-DNA complex by actively forcing specific allosteric structural changes within the central core DBD itself.	[50]
DBD	Dynamic networks and community analysis via MD simulations.	Computational	Applying network theory to the p53 core domain reveals a highly cohesive 38-member network of motionally covarying residues (“MD sectors”) that forms a single dense, interconnected component with an exceptionally high clustering coefficient (~0.73). This network preserves redundant long-range pathways for allosteric signaling. In silico alanine mutation of critical network hubs (such as R158 or L264) physically breaks these conduits, fundamentally disrupting global motional undercurrents and shifting the protein into distinct, non-native conformational substates within Markov State Models.	[51]

**Table 4:** Thermodynamic affinity (*K*_d_) of the p53 tetramer for diverse genomic response elements. The table quantifies the dissociation constants (in nM) for established p53 target promoters, categorized by their corresponding downstream cellular programs (attractor states). The marked disparity in *K*_d_ values between high-affinity sites driving cell cycle arrest or DNA repair (e.g., *p21*, *p53R2*) and low-affinity apoptotic sites (e.g., *BAX*, *PUMA BS1*) establishes the biochemical foundation of the Layer 5A transcriptomic pathway. This affinity gradient dictates a prioritized, concentration-dependent execution of biological programs, ensuring that irreversible apoptotic decisions remain strictly threshold-gated.

Cell Response (Attractor)	Element (Gene)	*K*_d_ (nM)
Negative regulator	*MDM2*	12.3 ± 2.9
Cell cycle arrest	*p21 5′site*	4.9 ± 0.6
	*p21 3′site*	12.0 ± 7.0
	*14-3-3s site 1*	9.9 ± 2.1
	*14-3-3s site 2*	10.0 ± 2.1
	*Cyclin G*	7.8 ± 1.6
DNA repair	*p53R2*	5.7 ± 0.8
	*PCNA*	6.6 ± 1.4
	*GADD45*	7.7 ± 1.2
Apoptosis	*PUMA BS1*	260 ± 50
	*PUMA BS2*	7.1 ± 1.8
	*Noxa*	8.6 ± 1.5
	*p53AIP1*	11.0 ± 1.8
	*p53DINP1*	98 ± 23
	*PIDD*	14.7 ± 5.0
	*Bax*	73 ± 33
	*IGF-BP3 Box A*	81 ± 25
	*P2XM*	221 ± 41

**Table 5:** Application of SOLID software design principles to the p53 signaling network architecture.

Principle	Definition (Robert C. Martin)	Implementation in the p53 Network	Cellular Consequences
S (Single Responsibility Principle)	Every software component should have only one responsibility or a single reason to change.	Extreme multitasking. p53 simultaneously governs four fundamental and often conflicting cellular programs: cell cycle arrest, apoptosis induction, DNA repair, and metabolic homeostasis.	Critical Single Point of Failure (SPOF). Because responsibility is undivided, any single missense mutation in the DNA-Binding Domain (DBD) instantly paralyzes all independent defense circuits, depriving the cell of the ability to execute either survival or self-elimination scenarios.
O (Open/Closed Principle)	Software entities should be open for extension but closed for modification (alteration of source code).	Core conservation with peripheral flexibility. The basal three-dimensional folding of the DBD is strictly conserved by evolution and protected from changes (“closed for modification”). Conversely, the signaling network is easily scalable (“open for extension”) through the recruitment of novel binding partners and the addition of PTM tags onto flexible terminal domains.	Evolutionary plasticity. This structural decoupling allows the cellular signaling system to rapidly adapt to novel stress types without necessitating a radical structural or logical redesign of the core controller protein itself.
L (Liskov Substitution Principle)	Objects in a program should be replaceable with instances of their subtypes without altering the correctness of the system.	Functional interchangeability of homologs. The p63 and p73 proteins are evolutionary relatives of p53, sharing a similar domain architecture, including a conserved DBD. This allows them to recognize analogous genomic regulatory elements and partially assume control over p53-target genes.	Hardware redundancy. The presence of homologous proteins establishes a protective topological buffer. Under specific physiological conditions, or upon the loss of p53, p63 and p73 can compensate for the regulatory deficit, preventing an immediate systemic collapse of the cell’s defense circuits.
I (Interface Segregation Principle)	Many client-specific interfaces are better than one general-purpose interface; components should not depend on interfaces they do not use.	Segregation of input and output ports. p53 utilizes distinct, structurally segregated autonomous domains (e.g., TAD, OD, PRD) to process incoming upstream signals and execute outgoing downstream commands.	Precise pathway selectivity. Specialized molecular interfaces allow the cell to accurately partition information streams. The controller can trigger an isolated program (e.g., cell cycle arrest) without inadvertently activating the terminal apoptotic cascade, safeguarding against false-positive lethal responses.
D (Dependency Inversion Principle)	High-level modules should not depend on low-level modules; both should depend on abstractions (interfaces).	Indirect control via a modification code. The central decision-making hub (p53) is isolated from direct physical contact with peripheral stress sensors. Instead, it responds to a universal “interface”—the regulatory code of post-translational modifications (PTMs)—generated by intermediate signaling cascades (e.g., ATM/ATR kinases).	Decoupling and network reliability. The kinase abstraction layer physically decouples the central p53 processor from the sensory periphery. Consequently, a malfunction in a single peripheral sensor does not compromise the global capacity of p53 to receive inputs from alternative circuits.

**Table 6:** Stratification of p53-targeted pharmacological strategies based on the hierarchical network architecture.

Architectural Layer	Therapeutic Strategy	Systems Engineering Mechanism	Systemic Limitations & Model Constraints
Layer 2: Local Protein–Protein Interaction	Direct Antagonists and Degraders (Directions 1 & 2)	Steric blockade of the binding interface (e.g., MDM2/X inhibitors) or targeted degradation to prevent the destruction of the central hub.	Ignores network topology. Induces systemic toxicity, hyperactivation of compensatory feedback loops, and rapid selection of resistant clones.
Layers 1 & 2: Post-Translational Code and Autonomous Domains	Pharmacological Chaperones (Direction 3)	Direct “hardware repair” of a local structural defect (e.g., the Y220C pocket) to restore the physical geometry of the DNA-reading interface.	Restores the physical interface (Layer 2) but does not compensate for the missing chemical PTM code (Layer 1), often leaving the signal incomplete.
Layer 3: Allosteric Integration	Allosteric Modulators (e.g., PK11000) (Direction 8)	Remote restoration of the global conformation of the full-length protein without direct interference at the primary active site.	Requires massive computational resources and advanced in silico algorithms to identify hidden allosteric signal transduction pathways.
Layer 4: Quaternary Assembly and Hardware Redundancy	Activation of Evolutionary Homologs (p63/p73) (Direction 4)	Rerouting data flow through redundant backup nodes possessing a native reading interface upon irreversible failure of the primary processor.	High risk of backup node sequestration into mutant hub aggregates (co-aggregation) or their suppression by dominant-negative isoforms (ΔNp63/ΔNp73).
Layer 5: Parallel Routing (The Mitochondrial Filter)	Synergistic Activation (Direction 5)	Topological lowering of the energetic resistance barrier at the system’s output (Mcl-1/Bcl-2), allowing a weak upstream pulse to breach the defense.	Establishes a secondary point of failure (vulnerability of mitochondrial effectors to acquired resistance mutations) and elevates systemic toxicity.
Layer 6: Biological Processes (Checkpoints)	Targeting Redundant Networks (Direction 6)	Blockade of the backup G2/M control mechanism (WEE1) upon which the tumor relies following the loss of the G1/S checkpoint, leading to fatal mitotic catastrophe.	Effective only in cells with an already broken central hub; may require synchronization with auxiliary sensitizing agents. Potential for systemic toxicity.
Layer 7: Global Cellular Context	Pharmacological Sheltering (Direction 7)	Temporary, forced deactivation of the central switchboard (p53) to shelter the healthy macro-system from a fatal response to therapeutic noise.	Not applicable for tumor eradication; utilized exclusively as a pharmacological shield to protect healthy tissues.

**Table 7:** Translational landscape of p53-targeted network interventions, highlighting the required molecular context, clinical status, and predominant resistance/toxicity mechanisms for key pharmacological agents.

Strategy/Agent(s)	Molecular Target	Required *TP53* Genotype	Experimental Level	Tumor Model (Examples)	Clinical Status	Major Toxicity/Resistance Mechanism	Key Ref.
Direct MDM2 Antagonists (Nutlin-3a, Idasanutlin)	MDM2 hydrophobic pocket	Wild-type	Preclinical/Clinical	AML, liposarcoma, solid tumors	Phase I–III	Severe thrombocytopenia, transition to senescence (SASP), acquired *TP53* mutations	[42,103,106]
Targeted Protein Degraders (MDM2 PROTACs)	MDM2 (recruiting E3 ligases)	Wild-type	Preclinical/Early Clinical	Leukemias, breast cancer	Preclinical/Phase I	Incomplete transcriptional signal, potential induction of alternative senescence attractors	[108,110,114]
Pocket-Specific Chaperones (Rezatapopt/ PC14586)	p53 Y220C structural crevice	Mutant (*TP53* Y220C strictly)	Preclinical/Clinical	Solid tumors, AML	Phase I/II	Clonal evolution (expansion of non-Y220C mutant clones), lack of afferent PTM signal	[115,116,117]
Covalent Chaperones (Eprenetapopt/APR-246)	Mutant p53 core domain cysteines	Mutant (Missense)	Preclinical/Clinical	MDS, AML	Phase II/III	Clonal escape, off-target covalent binding toxicity	[118,119]
Homolog Liberators (e.g., RETRA)	Mutant p53/p73 aggregate complex	Mutant (Gain-of-Function)	Preclinical (in vitro/in vivo)	Various solid tumors	Preclinical	Viral degradation of p73, stoichiometric buffering by ΔNp73 isoforms	[122,124]
Synergistic Activation (MDM2i + Venetoclax)	MDM2 and Bcl-2	Wild-type	Preclinical/Clinical	AML	Phase I/II	Acquired resistance via downstream mitochondrial effectors (e.g., *BAX* mutations), cumulative systemic toxicity	[125,126]
Targeting Redundant Nodes (Adavosertib/MK-1775)	WEE1 kinase (G2/M checkpoint)	Null/Mutant	Preclinical/Clinical	Ovarian, solid tumors	Phase II	Systemic toxicity against highly proliferative healthy tissues, activation of parallel bypass pathways (ATR/CHK1)	[127,128]
Pharmacological Sheltering (Pifithrin-α)	Wild-type p53	Wild-type (in healthy tissue)	Preclinical	N/A (Normal tissue protection)	Preclinical	Theoretical risk of long-term genomic instability or *de novo* carcinogenesis upon prolonged use	[129]

## Data Availability

Dataset available on request from the author.

## Abbreviations

The following abbreviations are used in this manuscript:

Ca	Afferent Coupling
CTD	C-Terminal Domain
DBD	DNA-Binding Domain
dCNA	Differential Contact Network Analysis
ECI	Early Contact Inhibition
GoF	Gain-of-Function
HCA	Heat Core Analysis
HMW-HA	High-Molecular-Mass Hyaluronic Acid
IDP	Intrinsically Disordered Protein
IDR	Intrinsically Disordered Region
*K*_d_	Dissociation Constant
MD	Molecular Dynamics
MDM2/MDMX	Murine Double Minute 2/X
MoRF	Molecular Recognition Feature
OD	Oligomerization Domain
PFT-α	Pifithrin-alpha
PPI	Protein–Protein Interaction
PRD	Proline-Rich Domain
PROTAC	Proteolysis Targeting Chimera
PTM	Post-Translational Modification
RE	Response Element
SASP	Senescence-Associated Secretory Phenotype
SBDD	Structure-Based Drug Design
SoC	System-on-a-Chip
SOLID	Single responsibility, Open-closed, Liskov substitution, Interface segregation, and Dependency inversion principles
SPOF	Single Point of Failure
TAD	Transactivation Domain

## References

[1] Wang S., Chen F.-E. (2022). Small-Molecule MDM2 Inhibitors in Clinical Trials for Cancer Therapy. Eur. J. Med. Chem..

[2] Halabi N., Rivoire O., Leibler S., Ranganathan R. (2009). Protein Sectors: Evolutionary Units of Three-Dimensional Structure. Cell.

[3] Barabási A.-L., Gulbahce N., Loscalzo J. (2011). Network Medicine: A Network-Based Approach to Human Disease. Nat. Rev. Genet..

[4] Belyi V.A., Ak P., Markert E., Wang H., Hu W., Puzio-Kuter A., Levine A.J. (2010). The Origins and Evolution of the P53 Family of Genes. Cold Spring Harb. Perspect. Biol..

[5] Åberg E., Saccoccia F., Grabherr M., Ore W.Y.J., Jemth P., Hultqvist G. (2017). Evolution of the P53-MDM2 Pathway. BMC Evol. Biol..

[6] Nedelcu A.M., Tan C. (2007). Early Diversification and Complex Evolutionary History of the P53 Tumor Suppressor Gene Family. Dev. Genes Evol..

[7] Joerger A.C., Wilcken R., Andreeva A. (2014). Tracing the Evolution of the P53 Tetramerization Domain. Structure.

[8] Levine A.J. (2020). P53: 800 Million Years of Evolution and 40 Years of Discovery. Nat. Rev. Cancer.

[9] Momand J., Villegas A., Belyi V.A. (2011). The Evolution of MDM2 Family Genes. Gene.

[10] Lee S.W., Seong M.W., Jeon Y.J., Chung C.H. (2012). Ubiquitin E3 Ligases Controlling P53 Stability. Anim. Cells Syst..

[11] Gao M.X., Liao E.H., Yu B., Wang Y., Zhen M., Derry W.B. (2008). The SCFFSN-1 Ubiquitin Ligase Controls Germline Apoptosis through CEP-1/P53 in C. Elegans. Cell Death Differ..

[12] Strasser A., Vaux D.L. (2018). Viewing BCL2 and Cell Death Control from an Evolutionary Perspective. Cell Death Differ..

[13] Banjara S., Suraweera C.D., Hinds M.G., Kvansakul M. (2020). The Bcl-2 Family: Ancient Origins, Conserved Structures, and Divergent Mechanisms. Biomolecules.

[14] Ha J.-H., Won E.-Y., Shin J.-S., Jang M., Ryu K.-S., Bae K.-H., Park S.G., Park B.C., Yoon H.S., Chi S.-W. (2011). Molecular Mimicry-Based Repositioning of Nutlin-3 to Anti-Apoptotic Bcl-2 Family Proteins. J. Am. Chem. Soc..

[15] Shin J.-S., Ha J.-H., He F., Muto Y., Ryu K.-S., Yoon H.S., Kang S., Park S.G., Park B.C., Choi S.-U. (2012). Structural Insights into the Dual-Targeting Mechanism of Nutlin-3. Biochem. Biophys. Res. Commun..

[16] Vaseva A.V., Moll U.M. (2009). The Mitochondrial P53 Pathway. Biochim. Biophys. Acta BBA-Bioenerg..

[17] Zhang Q., Lu H. (2010). Nutlin’s Two Roads toward Apoptosis. Cancer Biol. Ther..

[18] Barabási A.-L., Albert R. (1999). Emergence of Scaling in Random Networks. Science.

[19] Brooks C.L., Gu W. (2006). P53 Ubiquitination: Mdm2 and Beyond. Mol. Cell.

[20] Kruse J.-P., Gu W. (2009). Modes of P53 Regulation. Cell.

[21] Meek D.W., Anderson C.W. (2009). Posttranslational Modification of P53: Cooperative Integrators of Function. Cold Spring Harb. Perspect. Biol..

[22] Olsson A., Manzl C., Strasser A., Villunger A. (2007). How Important Are Post-Translational Modifications in P53 for Selectivity in Target-Gene Transcription and Tumour Suppression?. Cell Death Differ..

[23] Toledo F., Wahl G.M. (2006). Regulating the P53 Pathway: In Vitro Hypotheses, in Vivo Veritas. Nat. Rev. Cancer.

[24] Shieh S.-Y., Ikeda M., Taya Y., Prives C. (1997). DNA Damage-Induced Phosphorylation of P53 Alleviates Inhibition by MDM2. Cell.

[25] Gu W., Roeder R.G. (1997). Activation of P53 Sequence-Specific DNA Binding by Acetylation of the P53 C-Terminal Domain. Cell.

[26] Haupt Y., Maya R., Kazaz A., Oren M. (1997). Mdm2 Promotes the Rapid Degradation of P53. Nature.

[27] Chuikov S., Kurash J.K., Wilson J.R., Xiao B., Justin N., Ivanov G.S., McKinney K., Tempst P., Prives C., Gamblin S.J. (2004). Regulation of P53 Activity through Lysine Methylation. Nature.

[28] Huang J., Perez-Burgos L., Placek B.J., Sengupta R., Richter M., Dorsey J.A., Kubicek S., Opravil S., Jenuwein T., Berger S.L. (2006). Repression of P53 Activity by Smyd2-Mediated Methylation. Nature.

[29] Yang W.H., Kim J.E., Nam H.W., Ju J.W., Kim H.S., Kim Y.S., Cho J.W. (2006). Modification of P53 with O-Linked N-Acetylglucosamine Regulates P53 Activity and Stability. Nat. Cell Biol..

[30] Carter S., Bischof O., Dejean A., Vousden K.H. (2007). C-Terminal Modifications Regulate MDM2 Dissociation and Nuclear Export of P53. Nat. Cell Biol..

[31] Rodriguez M.S., Desterro J.M.P., Lain S., Midgley C.A., Lane D.P., Hay R.T. (1999). SUMO-1 Modification Activates the Transcriptional Response of P53. EMBO J..

[32] Xirodimas D.P., Saville M.K., Bourdon J.-C., Hay R.T., Lane D.P. (2004). Mdm2-Mediated NEDD8 Conjugation of P53 Inhibits Its Transcriptional Activity. Cell.

[33] Abida W.M., Nikolaev A., Zhao W., Zhang W., Gu W. (2007). FBXO11 Promotes the Neddylation of P53 and Inhibits Its Transcriptional Activity. J. Biol. Chem..

[34] Chothia C. (1984). Principles That Determine the Structure of Proteins. Annu. Rev. Biochem..

[35] Richardson J.S. (1981). The Anatomy and Taxonomy of Protein Structure. Adv. Protein Chem..

[36] Wetlaufer D.B. (1973). Nucleation, Rapid Folding, and Globular Intrachain Regions in Proteins. Proc. Natl. Acad. Sci. USA.

[37] Freedman D.A., Epstein C.B., Roth J.C., Levine A.J. (1997). A Genetic Approach to Mapping the P53 Binding Site in the MDM2 Protein. Mol. Med..

[38] Joerger A.C., Fersht A.R. (2008). Structural Biology of the Tumor Suppressor P53. Annu. Rev. Biochem..

[39] Cho Y., Gorina S., Jeffrey P.D., Pavletich N.P. (1994). Crystal Structure of a P53 Tumor Suppressor-DNA Complex: Understanding Tumorigenic Mutations. Science.

[40] Jeffrey P.D., Gorina S., Pavletich N.P. (1995). Crystal Structure of the Tetramerization Domain of the P53 Tumor Suppressor at 1.7 Angstroms. Science.

[41] Walker K.K., Levine A.J. (1996). Identification of a Novel P53 Functional Domain That Is Necessary for Efficient Growth Suppression. Proc. Natl. Acad. Sci. USA.

[42] Vassilev L.T., Vu B.T., Graves B., Carvajal D., Podlaski F., Filipovic Z., Kong N., Kammlott U., Lukacs C., Klein C. (2004). In Vivo Activation of the P53 Pathway by Small-Molecule Antagonists of MDM2. Science.

[43] Bykov V.J.N., Issaeva N., Shilov A., Hultcrantz M., Pugacheva E., Chumakov P., Bergman J., Wiman K.G., Selivanova G. (2002). Restoration of the Tumor Suppressor Function to Mutant P53 by a Low-Molecular-Weight Compound. Nat. Med..

[44] Degtjarik O., Golovenko D., Diskin-Posner Y., Abrahmsén L., Rozenberg H., Shakked Z. (2021). Structural Basis of Reactivation of Oncogenic P53 Mutants by a Small Molecule: Methylene Quinuclidinone (MQ). Nat. Commun..

[45] McKinney K., Mattia M., Gottifredi V., Prives C. (2004). P53 Linear Diffusion along DNA Requires Its C Terminus. Mol. Cell.

[46] Natan E., Baloglu C., Pagel K., Freund S.M.V., Morgner N., Robinson C.V., Fersht A.R., Joerger A.C. (2011). Interaction of the P53 DNA-Binding Domain with Its N-Terminal Extension Modulates the Stability of the P53 Tetramer. J. Mol. Biol..

[47] Sun X., Dyson H.J., Wright P.E. (2021). A Phosphorylation-Dependent Switch in the Disordered P53 Transactivation Domain Regulates DNA Binding. Proc. Natl. Acad. Sci. USA.

[48] Fadda E., Nixon M.G. (2017). The Transient Manifold Structure of the P53 Extreme C-Terminal Domain: Insight into Disorder, Recognition, and Binding Promiscuity by Molecular Dynamics Simulations. Phys. Chem. Chem. Phys. PCCP.

[49] Ning S., Zeng C., Wang H., Zhang J., Xue Y., Zhao Y. (2026). The Allosteric Regulation of the DNA-Binding Domain of P53 by the Intrinsically Disordered C-Terminal Domain. Pharmaceuticals.

[50] Laptenko O., Shiff I., Freed-Pastor W., Zupnick A., Mattia M., Freulich E., Shamir I., Kadouri N., Kahan T., Manfredi J. (2015). The P53 C Terminus Controls Site-Specific DNA Binding and Promotes Structural Changes within the Central DNA Binding Domain. Mol. Cell.

[51] Fabry J.D., Thayer K.M. (2023). Network Analysis of Molecular Dynamics Sectors in the P53 Protein. ACS Omega.

[52] Csete M., Doyle J. (2004). Bow Ties, Metabolism and Disease. Trends Biotechnol..

[53] Kitano H. (2004). Biological Robustness. Nat. Rev. Genet..

[54] Hartwell L.H., Hopfield J.J., Leibler S., Murray A.W. (1999). From Molecular to Modular Cell Biology. Nature.

[55] Weinberg R.L., Veprintsev D.B., Fersht A.R. (2004). Cooperative Binding of Tetrameric P53 to DNA. J. Mol. Biol..

[56] Wu X., Bayle J.H., Olson D., Levine A.J. (1993). The P53-Mdm-2 Autoregulatory Feedback Loop. Genes Dev..

[57] Michael D., Oren M. (2003). The P53-Mdm2 Module and the Ubiquitin System. Semin. Cancer Biol..

[58] Rajagopalan S., Huang F., Fersht A.R. (2011). Single-Molecule Characterization of Oligomerization Kinetics and Equilibria of the Tumor Suppressor P53. Nucleic Acids Res..

[59] Szklarczyk D., Nastou K., Koutrouli M., Kirsch R., Mehryary F., Hachilif R., Hu D., Peluso M.E., Huang Q., Fang T. (2025). The STRING Database in 2025: Protein Networks with Directionality of Regulation. Nucleic Acids Res..

[60] Weinberg R.L., Veprintsev D.B., Bycroft M., Fersht A.R. (2005). Comparative Binding of P53 to Its Promoter and DNA Recognition Elements. J. Mol. Biol..

[61] Veprintsev D.B., Fersht A.R. (2008). Algorithm for Prediction of Tumour Suppressor P53 Affinity for Binding Sites in DNA. Nucleic Acids Res..

[62] McLure K.G., Lee P.W. (1998). How P53 Binds DNA as a Tetramer. EMBO J..

[63] Kastenhuber E.R., Lowe S.W. (2017). Putting P53 in Context. Cell.

[64] Chipuk J.E., Kuwana T., Bouchier-Hayes L., Droin N.M., Newmeyer D.D., Schuler M., Green D.R. (2004). Direct Activation of Bax by P53 Mediates Mitochondrial Membrane Permeabilization and Apoptosis. Science.

[65] Kracikova M., Akiri G., George A., Sachidanandam R., Aaronson S.A. (2013). A Threshold Mechanism Mediates P53 Cell Fate Decision between Growth Arrest and Apoptosis. Cell Death Differ..

[66] Lahav G., Maroto M., Monk N.A.M. (2009). Oscillations by the P53-Mdm2 Feedback Loop. Cellular Oscillatory Mechanisms.

[67] Lev Bar-Or R., Maya R., Segel L.A., Alon U., Levine A.J., Oren M. (2000). Generation of Oscillations by the P53-Mdm2 Feedback Loop: A Theoretical and Experimental Study. Proc. Natl. Acad. Sci. USA.

[68] Lahav G., Rosenfeld N., Sigal A., Geva-Zatorsky N., Levine A.J., Elowitz M.B., Alon U. (2004). Dynamics of the P53-Mdm2 Feedback Loop in Individual Cells. Nat. Genet..

[69] Purvis J.E., Karhohs K.W., Mock C., Batchelor E., Loewer A., Lahav G. (2012). P53 Dynamics Control Cell Fate. Science.

[70] Huang S., Eichler G., Bar-Yam Y., Ingber D.E. (2005). Cell Fates as High-Dimensional Attractor States of a Complex Gene Regulatory Network. Phys. Rev. Lett..

[71] Choi M., Shi J., Jung S.H., Chen X., Cho K.-H. (2012). Attractor Landscape Analysis Reveals Feedback Loops in the P53 Network That Control the Cellular Response to DNA Damage. Sci. Signal..

[72] Zhang X.-P., Liu F., Wang W. (2011). Two-Phase Dynamics of P53 in the DNA Damage Response. Proc. Natl. Acad. Sci. USA.

[73] Xiong W., Ferrell J.E. (2003). A Positive-Feedback-Based Bistable “memory Module” That Governs a Cell Fate Decision. Nature.

[74] Tyson J.J., Chen K.C., Novak B. (2003). Sniffers, Buzzers, Toggles and Blinkers: Dynamics of Regulatory and Signaling Pathways in the Cell. Curr. Opin. Cell Biol..

[75] Bak P., Tang C., Wiesenfeld K. (1987). Self-Organized Criticality: An Explanation of the 1/f Noise. Phys. Rev. Lett..

[76] Kauffman S.A. (1993). The Origins of Order: Self-Organization and Selection in Evolution.

[77] Haldeman C., Beggs J.M. (2005). Critical Branching Captures Activity in Living Neural Networks and Maximizes the Number of Metastable States. Phys. Rev. Lett..

[78] Kinouchi O., Copelli M. (2006). Optimal Dynamical Range of Excitable Networks at Criticality. Nat. Phys..

[79] Nykter M., Price N.D., Aldana M., Ramsey S.A., Kauffman S.A., Hood L.E., Yli-Harja O., Shmulevich I. (2008). Gene Expression Dynamics in the Macrophage Exhibit Criticality. Proc. Natl. Acad. Sci. USA.

[80] Mora T., Bialek W. (2011). Are Biological Systems Poised at Criticality?. J. Stat. Phys..

[81] Martin R. (1994). OO Design Quality Metrics. Anal. Depend..

[82] Martin R. (2003). Agile Software Development: Principles, Patterns, and Practices.

[83] Barabási A.-L., Oltvai Z.N. (2004). Network Biology: Understanding the Cell’s Functional Organization. Nat. Rev. Genet..

[84] Albert R., Jeong H., Barabási A.-L. (2000). Error and Attack Tolerance of Complex Networks. Nature.

[85] Tu Y. (2000). How Robust Is the Internet?. Nature.

[86] Baugh E.H., Ke H., Levine A.J., Bonneau R.A., Chan C.S. (2018). Why Are There Hotspot Mutations in the TP53 Gene in Human Cancers?. Cell Death Differ..

[87] Chen X., Zhang T., Su W., Dou Z., Zhao D., Jin X., Lei H., Wang J., Xie X., Cheng B. (2022). Mutant P53 in Cancer: From Molecular Mechanism to Therapeutic Modulation. Cell Death Dis..

[88] Patil M.R., Bihari A. (2025). Cancer Classification Through P53 Hotspot Mutations: An Ensemble Learning Approach. Cell Biol. Int..

[89] Attardi L.D., Boutelle A.M. (2024). Targeting P53 Gain-of-Function Activity in Cancer Therapy: A Cautionary Tale. Cell Death Differ..

[90] Alvarado-Ortiz E., de la Cruz-López K.G., Becerril-Rico J., Sarabia-Sánchez M.A., Ortiz-Sánchez E., García-Carrancá A. (2021). Mutant P53 Gain-of-Function: Role in Cancer Development, Progression, and Therapeutic Approaches. Front. Cell Dev. Biol..

[91] Zhang C., Liu J., Xu D., Zhang T., Hu W., Feng Z. (2020). Gain-of-Function Mutant P53 in Cancer Progression and Therapy. J. Mol. Cell Biol..

[92] Lozano G., Prives C., Sabapathy K. (2025). Mutant P53 Gain of Function: Why Many See It, Why Some Do Not. Cancer Discov..

[93] Wang H., Guo M., Wei H., Chen Y. (2023). Targeting P53 Pathways: Mechanisms, Structures and Advances in Therapy. Signal Transduct. Target. Ther..

[94] Peuget S., Zhou X., Selivanova G. (2024). Translating P53-Based Therapies for Cancer into the Clinic. Nat. Rev. Cancer.

[95] Cheok C.F., Verma C.S., Baselga J., Lane D.P. (2011). Translating P53 into the Clinic. Nat. Rev. Clin. Oncol..

[96] Kussie P.H., Gorina S., Marechal V., Elenbaas B., Moreau J., Levine A.J., Pavletich N.P. (1996). Structure of the MDM2 Oncoprotein Bound to the P53 Tumor Suppressor Transactivation Domain. Science.

[97] Bazanov D.R., Lozinskaya N.A. (2025). The Evolution of Nutlins as P53-MDM2 Inhibitors. Med. Chem. Res..

[98] Tovar C., Rosinski J., Filipovic Z., Higgins B., Kolinsky K., Hilton H., Zhao X., Vu B.T., Qing W., Packman K. (2006). Small-Molecule MDM2 Antagonists Reveal Aberrant P53 Signaling in Cancer: Implications for Therapy. Proc. Natl. Acad. Sci. USA.

[99] Huang Y., Che X., Wang P.W., Qu X. (2024). P53/MDM2 Signaling Pathway in Aging, Senescence and Tumorigenesis. Semin. Cancer Biol..

[100] Gannon H.S., Donehower L.A., Lyle S., Jones S.N. (2011). Mdm2–P53 Signaling Regulates Epidermal Stem Cell Senescence and Premature Aging Phenotypes in Mouse Skin. Dev. Biol..

[101] Kitagawa M., Aonuma M., Lee S.H., Fukutake S., McCormick F. (2008). E2F-1 Transcriptional Activity Is a Critical Determinant of Mdm2 Antagonist-Induced Apoptosis in Human Tumor Cell Lines. Oncogene.

[102] Wu D., Prives C. (2018). Relevance of the P53–MDM2 Axis to Aging. Cell Death Differ..

[103] Shen H., Maki C.G. (2010). Persistent P21 Expression after Nutlin-3a Removal Is Associated with Senescence-like Arrest in 4N Cells. J. Biol. Chem..

[104] Coppé J.-P., Patil C.K., Rodier F., Sun Y., Muñoz D.P., Goldstein J., Nelson P.S., Desprez P.-Y., Campisi J. (2008). Senescence-Associated Secretory Phenotypes Reveal Cell-Nonautonomous Functions of Oncogenic RAS and the P53 Tumor Suppressor. PLoS Biol..

[105] Wang B., Han J., Elisseeff J.H., Demaria M. (2024). The Senescence-Associated Secretory Phenotype and Its Physiological and Pathological Implications. Nat. Rev. Mol. Cell Biol..

[106] Khoo K.H., Verma C.S., Lane D.P. (2014). Drugging the P53 Pathway: Understanding the Route to Clinical Efficacy. Nat. Rev. Drug Discov..

[107] Wang B., Wu S., Liu J., Yang K., Xie H., Tang W. (2019). Development of Selective Small Molecule MDM2 Degraders Based on Nutlin. Eur. J. Med. Chem..

[108] Vicente A.T.S., Salvador J.A.R. (2022). MDM2-Based Proteolysis-Targeting Chimeras (PROTACs): An Innovative Drug Strategy for Cancer Treatment. Int. J. Mol. Sci..

[109] Li H., Cai X., Yang X., Zhang X. (2024). An Overview of PROTACs Targeting MDM2 as a Novel Approach for Cancer Therapy. Eur. J. Med. Chem..

[110] Kandarpa M., Peterson L.F., Potu H., Ramappan M., Liu Y., Polk A., Wang S., Talpaz M. (2026). Activity of PROTAC MDM2 Degrader in Primary Leukemia Cells and PDX Models. Leukemia.

[111] Nietzold F., Rubner S., Labuzek B., Golik P., Surmiak E., del Corte X., Kitel R., Protzel C., Reppich-Sacher R., Stichel J. (2023). Nutlin-3a-Aa: Improving the Bioactivity of a P53/MDM2 Interaction Inhibitor by Introducing a Solvent-Exposed Methylene Group. ChemBioChem.

[112] Kim Y., Kim J.-W., Choi J., Kim J., Park S., Choi W., An H., Kim J., Kim M., Choi S. (2026). PROTAC-Mediated Targeted Degradation of MDM2 Induces Tumor-Suppressive Signaling in Osteosarcoma Cells. Cells.

[113] Jeong S., Cha J.-K., Ahmed W., Kim J., Kim M., Hong K.T., Choi W., Choi S., Yoo T.H., An H. (2025). Development of MDM2-Targeting PROTAC for Advancing Bone Regeneration. Adv. Sci..

[114] Adams C.M., Mitra R., Xiao Y., Michener P., Palazzo J., Chao A., Gour J., Cassel J., Salvino J.M., Eischen C.M. (2023). Targeted MDM2 Degradation Reveals a New Vulnerability for P53-Inactivated Triple-Negative Breast Cancer. Cancer Discov..

[115] Ma Z., Shen Q., Zhou J. (2025). Rezatapopt (PC14586): A First-in-Class Small Molecule P53 Y220C Mutant Protein Stabilizer in Clinical Trials. J. Med. Chem..

[116] Puzio-Kuter A.M., Xu L., McBrayer M.K., Dominique R., Li H.H., Fahr B.J., Brown A.M., Wiebesiek A.E., Russo B.M., Mulligan C.L. (2025). Restoration of the Tumor Suppressor Function of Y220C-Mutant P53 by Rezatapopt, a Small-Molecule Reactivator. Cancer Discov..

[117] Carter B.Z., Mak P.Y., Ayoub E., Wu X., Ke B., Nishida Y., Futreal A., Ostermann L.B., Bedoy A.D., Boettcher S. (2025). Restoring P53 Wild-Type Conformation in TP53-Y220C–Mutant Acute Myeloid Leukemia. Blood.

[118] Sallman D.A., DeZern A.E., Garcia-Manero G., Steensma D.P., Roboz G.J., Sekeres M.A., Cluzeau T., Sweet K.L., McLemore A., McGraw K.L. (2021). Eprenetapopt (APR-246) and Azacitidine in TP53-Mutant Myelodysplastic Syndromes. J. Clin. Oncol..

[119] Garcia-Manero G., Goldberg A.D., Winer E.S., Altman J.K., Fathi A.T., Odenike O., Roboz G.J., Sweet K., Miller C., Wennborg A. (2023). Eprenetapopt Combined with Venetoclax and Azacitidine in TP53-Mutated Acute Myeloid Leukaemia: A Phase 1, Dose-Finding and Expansion Study. Lancet Haematol..

[120] Wang Z., Hu H., Heitink L., Rogers K., You Y., Tan T., Suen C.L.W., Garnham A., Chen H., Lieschke E. (2023). The Anti-Cancer Agent APR-246 Can Activate Several Programmed Cell Death Processes to Kill Malignant Cells. Cell Death Differ..

[121] Osterburg C., Dötsch V. (2022). Structural Diversity of P63 and P73 Isoforms. Cell Death Differ..

[122] Cai B.-H., Hsu Y.-C., Yeh F.-Y., Lin Y.-R., Lu R.-Y., Yu S.-J., Shaw J.-F., Wu M.-H., Tsai Y.-Z., Lin Y.-C. (2022). P63 and P73 Activation in Cancers with P53 Mutation. Biomedicines.

[123] Klein A.M., Biderman L., Tong D., Alaghebandan B., Plumber S.A., Mueller H.S., van Vlimmeren A., Katz C., Prives C. (2021). MDM2, MDMX, and P73 Regulate Cell-Cycle Progression in the Absence of Wild-Type P53. Proc. Natl. Acad. Sci. USA.

[124] Kravchenko J.E., Ilyinskaya G.V., Komarov P.G., Agapova L.S., Kochetkov D.V., Strom E., Frolova E.I., Kovriga I., Gudkov A.V., Feinstein E. (2008). Small-Molecule RETRA Suppresses Mutant P53-Bearing Cancer Cells through a P73-Dependent Salvage Pathway. Proc. Natl. Acad. Sci. USA.

[125] Lehmann C., Friess T., Birzele F., Kiialainen A., Dangl M. (2016). Superior Anti-Tumor Activity of the MDM2 Antagonist Idasanutlin and the Bcl-2 Inhibitor Venetoclax in P53 Wild-Type Acute Myeloid Leukemia Models. J. Hematol. Oncol..

[126] Pan R., Ruvolo V., Mu H., Leverson J.D., Nichols G., Reed J.C., Konopleva M., Andreeff M. (2017). Synthetic Lethality of Combined Bcl-2 Inhibition and P53 Activation in AML: Mechanisms and Superior Antileukemic Efficacy. Cancer Cell.

[127] Thangaretnam K., Islam M.O., Lv J., El-Rifai A., Perloff A., Soutto H.L., Peng D., Chen Z. (2025). WEE1 Inhibition in Cancer Therapy: Mechanisms, Synergies, Preclinical Insights, and Clinical Trials. Crit. Rev. Oncol. Hematol..

[128] Clausse V., Goloudina A.R., Uyanik B., Kochetkova E.Y., Richaud S., Fedorova O.A., Hammann A., Bardou M., Barlev N.A., Garrido C. (2016). Wee1 Inhibition Potentiates Wip1-Dependent P53-Negative Tumor Cell Death during Chemotherapy. Cell Death Dis..

[129] Komarov P.G., Komarova E.A., Kondratov R.V., Christov-Tselkov K., Coon J.S., Chernov M.V., Gudkov A.V. (1999). A Chemical Inhibitor of P53 That Protects Mice from the Side Effects of Cancer Therapy. Science.

[130] Teoh P.J., Chung T.H., Sebastian S., Choo S.N., Yan J., Ng S.B., Fonseca R., Chng W.J. (2014). P53 Haploinsufficiency and Functional Abnormalities in Multiple Myeloma. Leukemia.

[131] Stühmer T., Chatterjee M., Hildebrandt M., Herrmann P., Gollasch H., Gerecke C., Theurich S., Cigliano L., Manz R.A., Daniel P.T. (2005). Nongenotoxic Activation of the P53 Pathway as a Therapeutic Strategy for Multiple Myeloma. Blood.

[132] Tokalov S.V., Abolmaali N.D. (2010). Protection of P53 Wild Type Cells from Taxol by Nutlin-3 in the Combined Lung Cancer Treatment. BMC Cancer.

[133] Stine K.C., Wahl E.C., Liu L., Skinner R.A., VanderSchilden J., Bunn R.C., Montgomery C.O., Aronson J., Becton D.L., Nicholas R.W. (2016). Nutlin-3 Treatment Spares Cisplatin-Induced Inhibition of Bone Healing While Maintaining Osteosarcoma Toxicity. J. Orthop. Res. Off. Publ. Orthop. Res. Soc..

[134] Thayer K.M., Stetson S., Caballero F., Chiu C., Han I.S.M. (2024). Navigating the Complexity of P53-DNA Binding: Implications for Cancer Therapy. Biophys. Rev..

[135] Stetson S., Caballero Mancía F., Thayer K.M. (2026). Restoration of the Y220C P53 Full-Length Mutant by PK11000: A Molecular Dynamics Study of an Intrinsically Disordered Protein. ACS Omega.

[136] Yao X.-Q., Momin M., Hamelberg D. (2018). Elucidating Allosteric Communications in Proteins with Difference Contact Network Analysis. J. Chem. Inf. Model..

[137] García-Cao I., García-Cao M., Martín-Caballero J., Criado L.M., Klatt P., Flores J.M., Weill J., Blasco M.A., Serrano M. (2002). “Super P53” Mice Exhibit Enhanced DNA Damage Response, Are Tumor Resistant and Age Normally. EMBO J..

[138] Sulak M., Fong L., Mika K., Chigurupati S., Yon L., Mongan N.P., Emes R.D., Lynch V.J. (2016). TP53 Copy Number Expansion Is Associated with the Evolution of Increased Body Size and an Enhanced DNA Damage Response in Elephants. eLife.

[139] Seluanov A., Hine C., Azpurua J., Feigenson M., Bozzella M., Mao Z., Catania K.C., Gorbunova V. (2009). Hypersensitivity to Contact Inhibition Provides a Clue to Cancer Resistance of Naked Mole-Rat. Proc. Natl. Acad. Sci. USA.

[140] Tian X., Azpurua J., Hine C., Vaidya A., Myakishev-Rempel M., Ablaeva J., Mao Z., Nevo E., Gorbunova V., Seluanov A. (2013). High-Molecular-Mass Hyaluronan Mediates the Cancer Resistance of the Naked Mole Rat. Nature.

[141] Deuker M.M., Lewis K.N., Ingaramo M., Kimmel J., Buffenstein R., Settleman J. (2020). Unprovoked Stabilization and Nuclear Accumulation of the Naked Mole-Rat P53 Protein. Sci. Rep..

